# Insights in the Complex DegU, DegS, and Spo0A Regulation System of Paenibacillus polymyxa by CRISPR-Cas9-Based Targeted Point Mutations

**DOI:** 10.1128/aem.00164-22

**Published:** 2022-05-19

**Authors:** Meliawati Meliawati, Tobias May, Jeanette Eckerlin, Daniel Heinrich, Andrea Herold, Jochen Schmid

**Affiliations:** a Institute of Molecular Microbiology and Biotechnology, University of Münster, Münster, Germany; b Department of Biotechnology and Food Science, Norwegian University of Science and Technology, Trondheim, Norway; c BASF SE, Ludwigshafen am Rhein, Germany; North Carolina State University

**Keywords:** CRISPR-Cas9, *Paenibacillus polymyxa*, point mutations, regulatory proteins

## Abstract

Despite being unicellular organisms, bacteria undergo complex regulation mechanisms which coordinate different physiological traits. Among others, DegU, DegS, and Spo0A are the pleiotropic proteins which govern various cellular responses and behaviors. However, the functions and regulatory networks between these three proteins are rarely described in the highly interesting bacterium Paenibacillus polymyxa. In this study, we investigate the roles of DegU, DegS, and Spo0A by introduction of targeted point mutations facilitated by a CRISPR-Cas9-based system. In total, five different mutant strains were generated, the single mutants DegU Q218*, DegS L99F, and Spo0A A257V, the double mutant DegU Q218* DegS L99F, and the triple mutant DegU Q218* DegS L99F Spo0A A257V. Characterization of the wild-type and the engineered strains revealed differences in swarming behavior, conjugation efficiency, sporulation, and viscosity formation of the culture broth. In particular, the double mutant DegU Q218* DegS L99F showed a significant increase in conjugation efficiency as well as a stable exopolysaccharides formation. Furthermore, we highlight similarities and differences in the roles of DegU, DegS, and Spo0A between P. polymyxa and related species. Finally, this study provides novel insights into the complex regulatory system of P. polymyxa DSM 365.

**IMPORTANCE** To date, only limited knowledge is available on how complex cellular behaviors are regulated in P. polymyxa. In this study, we investigate several regulatory proteins which play a role in governing different physiological traits. Precise targeted point mutations were introduced to their respective genes by employing a highly efficient CRISPR-Cas9-based system. Characterization of the strains revealed some similarities, but also differences, to the model bacterium Bacillus subtilis with regard to the regulation of cellular behaviors. Furthermore, we identified several strains which have superior performance over the wild-type. The applicability of the CRISPR-Cas9 system as a robust genome editing tool, in combination with the engineered strain with increased genetic accessibility, would boost further research in P. polymyxa and support its utilization for biotechnological applications. Overall, our study provides novel insights, which will be of importance in understanding how multiple cellular processes are regulated in *Paenibacillus* species.

## INTRODUCTION

To survive changing environmental conditions, complex genetic signaling networks are involved in the control of cellular adaption processes in many bacteria. In B. subtilis, the two-component system DegS/DegU is an important regulator for various differentiation strategies, such as motility, formation of extracellular biofilm matrixes, variation of colony architecture, synthesis of degradative enzymes, and development of natural competence (1–3). Further adaption processes are controlled in Bacilli by the master regulator Spo0A, which coordinates the transition of growing cells to spores. Besides the initiation of sporulation, this multicomponent phosphorelay system regulates transcription, directly or indirectly, of more than 500 genes involved in the adaption to nutrient starvation and changing environmental conditions (4, 5). In combination, DegS/DegU and Spo0A are often found to either jointly or antagonistically control various adaptive traits. Morphological variations of colonies on agar plates, as well as the synthesis of exopolysaccharide (EPS) or degradative enzymes, such as subtilisin, were found to be strongly dependent on the respective level of phosphorelay of both regulator systems in B. subtilis (6–9).

Due to their multifunctionalities, DegU, DegS, and Spo0A have been widely used as prominent targets in academia and industry for the genetic optimization of Bacilli. For instance, mutations such as *degU32*(Hy) and *degS200*(Hy) were applied to stimulate synthesis of degradative enzymes with commercial significance (1). Moreover, deletions of the 15 C-terminal residues of Spo0A were used to block the early stage (0) of sporulation while keeping other Spo0A-regulated genes active (10). Prominently, substitutions of the alanine at amino acid position 257 (A257) to either valine or glutamic acid were used to generate a sporulation-deficient strain without impairing the Spo0A-mediated *abrB* promoter repression, thus enabling increased productivity of enzymes and antibiotics as well as a delayed entry into the stationary growth phase (11–13).

While much is known about the regulatory network controlled by DegU, DegS, and Spo0A in B. subtilis, only a little knowledge exists about their roles in *Paenibacillus*, even though this genus has gained an enormous interest for diverse biotechnological applications from production of enzymes and EPSs to antimicrobials and platform chemicals (14–18). This is, inter alia, due to the complex genetic accessibility of *Paenibacillus* (17, 19), which limits the number of publications so far testing targeted mutations within the DegS/DegU and Spo0A systems. Therefore, most reports are limited to untargeted mutations obtained from random mutagenesis approaches or by adaptive laboratory evolutions. As an example, Hou et al. (20) detected spontaneous mutations in Spo0A (R211H) and DegU (Q218R) at once in laboratory cultivations of the biocontrol strain Paenibacillus polymyxa SC2, which resulted in a more stable, partially transparent morphology that was also lacking the ability to form endospores. In another study, a transcriptomic analysis of the same strain revealed a strong upregulation of *degU* and a consecutive activation of biofilm- and EPS-related genes, such as *epsB*, *epsE*, and *abh*, during the colonization process of P. polymyxa SC2 in the rhizosphere of pepper plants (21). This is in accordance with a comparative genomic analysis between different P. polymyxa strains, suggesting a key role of DegS/DegU and Spo0A in the synthesis of EPSs, which, in turn, enables a motion and wide distribution of the strains and their antimicrobial metabolites over the plant (22). With regard to motility, so far, the only reported targeted knockout of *degS* in *Paenibacillus* resulted in a nonmotile strain as a consequence of a blocked flagellar gene transcription (23).

To understand the impact of the regulatory systems DegS/DegU and Spo0A on adaptive traits and growth of *Paenibacillus*, we employed a CRISPR-Cas9-based system for the introduction of targeted point mutations in the respective genes. As a representative strain, we have selected the industrial relevant strain P. polymyxa DSM 365, which has already been utilized for the production of tailor-made microbial biopolymers and 2,3-butanediol (15, 18). The A257V mutation in Spo0A was chosen based on its prominent role in B. subtilis physiology in order to identify the transferability to P. polymyxa. From this, a weakening of the promoter binding site by reduced homodimer formation or a decreased contact with the cognate sigma factors is expected, as observed in B. subtilis (11, 13). Based on the study by Hou et al. (20), which reported the double mutants of DegU and Spo0A, we have been inspired to integrate a DegU Q218* mutation in P. polymyxa, which mediates a premature truncation of DegU and thus potentially impairs its phosphorelay without fully abolishing its activity. In addition, DegS was modified in the DNA binding domain on position L99F, based on a report of decreased viscosity detected in a randomly mutagenized *Paenibacillus* strain having mutations in the DNA binding of DegS (24). Furthermore, to analyze the interaction of both regulatory systems and the impact of the mutations described, a double mutant, DegU Q218* DegS L99F, and a triple mutant, DegU Q218* DegS L99F Spo0A A257V, were generated. All engineered strains were evaluated with regard to their physiological traits as well as the changes in viscosity profile of the cultivation broth.

## RESULTS

### CRISPR-Cas9-based targeted point mutations.

**FIG 1:**
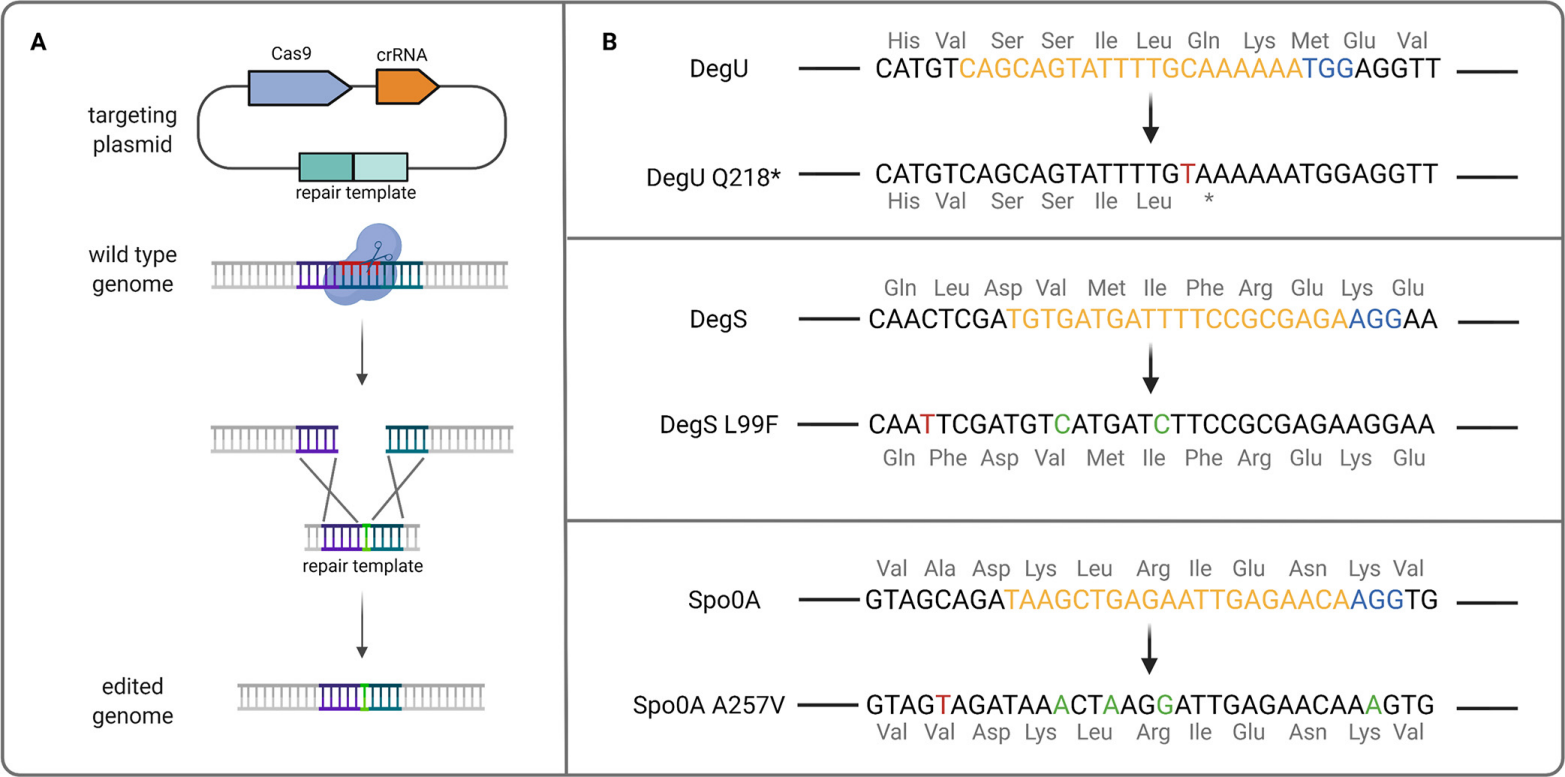
(A) Schematic overview of CRISPR-Cas9-mediated targeted point mutations. (B) Wild-type sequences of *degU*, *degS*, and *spo0A* genes of P. polymyxa (top) and the respective targeted mutations in this study (bottom). Mutations which caused DegU Q218*, DegS L99F, and Spo0A A257V are indicated in red. Additional silent mutations, which were added to increase the editing efficiency of the targeted point mutations, are indicated in green. Spacer sequences and the respective PAM sites are indicated in yellow and blue, respectively. The amino acid sequences of each protein are also indicated.

In this study, the regulatory genes *degU*, *degS*, and *spo0A* were chosen to evaluate their roles in regulating different physiological traits and EPSs formation in P. polymyxa DSM 365. For *degU*, a C→T substitution at nucleotide position 652 was introduced, which led to a DegU Q218* mutant. For *degS*, a C→T substitution was targeted at nucleotide position 295 to generate DegS L99F. Meanwhile, the targeted mutation for the *spo0A* was a C→T substitution at nucleotide position 770, which resulted in Spo0A A257V. Moreover, we combined the mutations to generate the double mutant DegU Q218* DegS L99F and the triple mutant DegU Q218* DegS L99F Spo0A A257V. To achieve this, the CRISPR-Cas9-based system was employed to mediate the introduction of the desired point mutations (Fig. 1A). The 20-nucleotide spacer sequence was selected based on the closest proximity to the targeted position. For DegU, the choice of a spacer sequence in which the targeted nucleotide was located was possible. However, this was not possible for DegS and Spo0A. Therefore, in addition to the originally targeted mutations, several silent mutations were introduced in the original positions of the spacer or the protospacer-adjacent motif (PAM) site in order to avoid Cas9 attacking the desired mutants (Fig. 1B). The editing efficiency varied between the different modifications. The editing efficiencies for the single mutants DegU Q218*, DegS L99F, and Spo0A A257V were 4/10 (40%), 1/20 (5%), and 9/10 (90%), respectively. Meanwhile, the double mutant DegU Q218* DegS L99F and triple mutant DegU Q218* DegS L99F Spo0A A257V were obtained with 1/10 (10%) and 2/2 (100%) editing efficiency, respectively. In this case, as observed for Spo0A A257V, it seems that point mutations in the PAM site, in combination with additional mutations in the spacer sequence, increased the overall editing efficiency. Finally, the employed system was able to introduce up to five mutations simultaneously and thus would be highly applicable for the generation of mutant strains with multiple targeted modifications.

### Sequence alignment and protein modeling.

**FIG 2:**
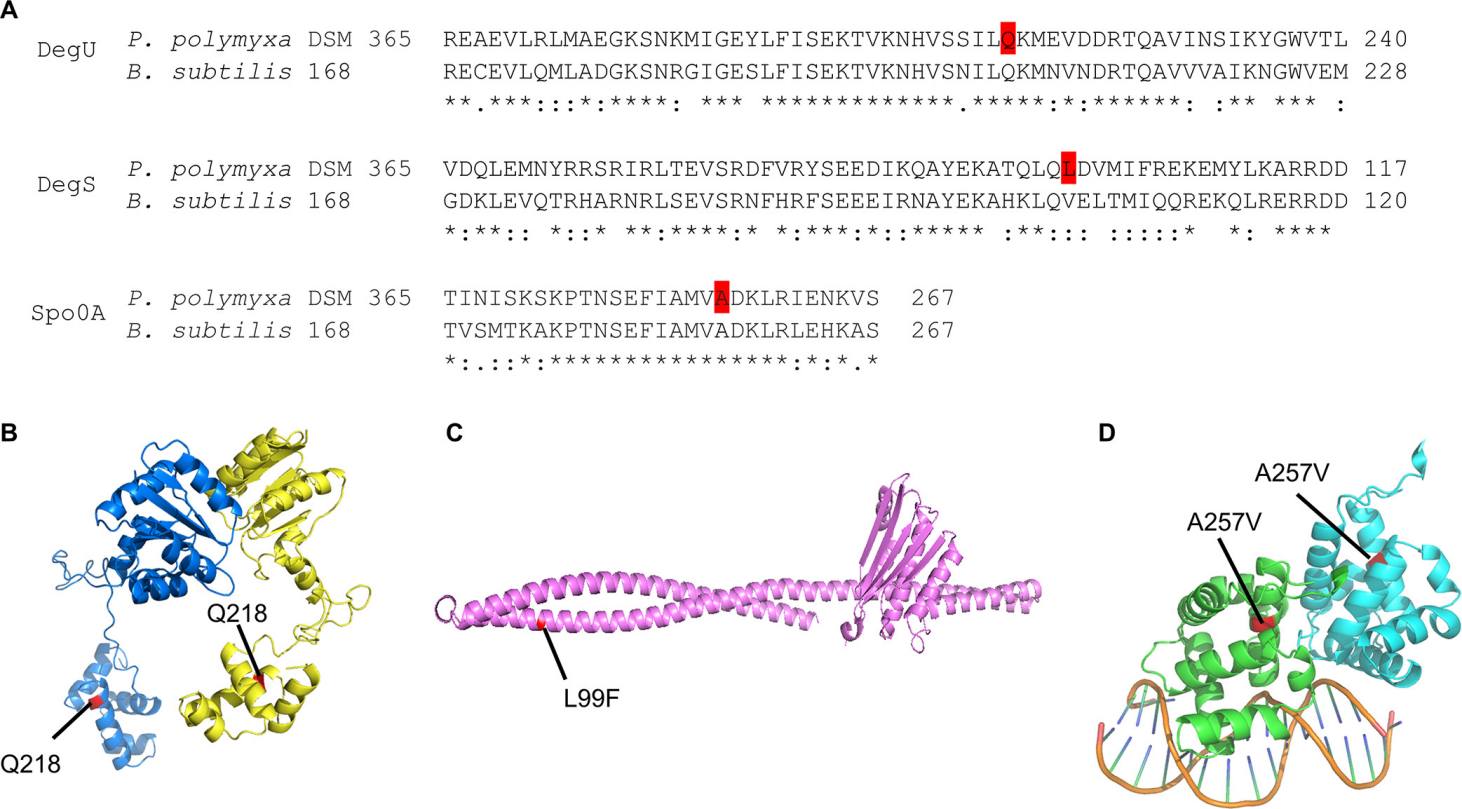
(A) Alignment of protein sequences of DegU, DegS, and Spo0A of P. polymyxa DSM 365 and B. subtilis 168. The targeted residues for the mutations investigated in this study are highlighted in red. Protein modeling of DegU (B), DegS L99F (C), and Spo0A A257V (D) of P. polymyxa DSM 365. DegU and Spo0A are shown in their dimer forms. For simplicity, only residues 141 to 267 are shown for Spo0A. The mutated residues are highlighted in red in the protein structure.

Little is known about the role of regulatory proteins in P. polymyxa. Therefore, we were intrigued to see whether the proteins investigated in this study portrayed sequence similarity with the model bacterium B. subtilis and if we can draw a functional correlation based on the sequence similarity, even though, for instance, *Paenibacillus* is lacking *degQ* and *degR*, which are required for efficient phosphotransfer from DegS~P to DegU and stable phosphorelay of DegU in B. subtilis (23, 25, 26). In general, both bacteria portrayed amino acid sequence similarities in their DegU, DegS, and Spo0A, with identities of 54%, 42%, and 69%, respectively. Interestingly, the targeted amino acids of DegU and Spo0A are among the conserved amino acids, which might hint at the essentiality of these residues. Meanwhile, the targeted L99 residue in DegS is not among the conserved amino acids but corresponds to its conservative counterparts, V102, in B. subtilis instead (Fig. 2A; see Fig. S2 in the supplemental material). In B. subtilis, DegU has two protein domains, the response regulatory domain (amino acids [aa] 5 to 121) and the DNA binding domain (aa 159 to 224) (27). The DNA binding domain of DegU is composed of a helix-turn-helix (HTH), which belongs to LuxR-type DNA binding domain. It is important to note that the DegU mutation in this study occurred in the DNA binding domain, which leads to a premature stop codon. As mutation of DegS does not take place within the histidine kinase domain (28), it is not expected that the mutation will lead to a significant change in its phosphorylation role. Meanwhile, the Spo0A mutation occurred in the C-terminal region, away from the response regulatory domain and the HTH binding domain.

To better understand the effect of the targeted mutations on the three-dimensional structure of the proteins, we performed modeling studies for the DegU, DegS, and Spo0A (Fig. 2B to D). The Q218* mutation results in a truncated version of DegU, which makes it lose one α-helix motif at the C terminus, which might be responsible for the recognition helix. Therefore, it is plausible that this mutation would affect the binding affinity of DegU to the promoter region of the different genes it modulates. On the other hand, the L99F mutation in the DegS occurs in the coiled-coil structure. As leucine is a strong helix-forming residue (29), its substitution for phenylalanine might weaken the helix structure in that region. Moreover, this mutation may cause a change in the phosphorylation level of the DegS itself, as observed from mutations in nearby locations (S76A or S76D) in B. subtilis (28). Similarly, the A257V mutation in Spo0A is proposed to affect the flexibility and orientation of the helix structure, thus weakening Spo0A dimerization (Fig. S3) (30).

### Swarming assay.

**FIG 3:**
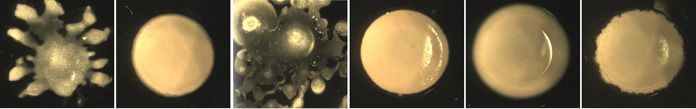
Evaluation of the swarming motility of wild-type and engineered mutant strains on LB plates containing 0.4% agar. From left to right are wild-type, DegU Q218*, DegS L99F, DegU Q218* DegS L99F, Spo0A A257V, and DegU Q218* DegS L99F Spo0A A257V.

It is beneficial for bacteria, in their natural habitat, to have the capability to move toward nutrients or away from harmful compounds, which can ensure their survival. Likewise, P. polymyxa is a flagellar-forming bacterium that is capable of two types of motility, swimming in liquid and swarming on a surface (31). Among the engineered strains in this study, only DegS L99F retained the swarming motility, while the other strains harboring mutations in DegU or Spo0A lost their swarming ability. Interestingly, the DegS L99F mutant seems to have higher swarming motility than the wild-type (Fig. 3). This provides a deeper understanding of the importance of DegU and Spo0A on modulated swarming behavior of P. polymyxa. This finding is in line with what was observed for B. subtilis, in which knockout experiments showed that DegU is essential for swarming motility, while DegS is not (32). Furthermore, the previously reported study by Barreto et al. (27) showed that a mutation at a nearby position (DegU I186M) also resulted in decreased swarming motility in B. subtilis.

### Conjugation efficiency.

**FIG 4:**
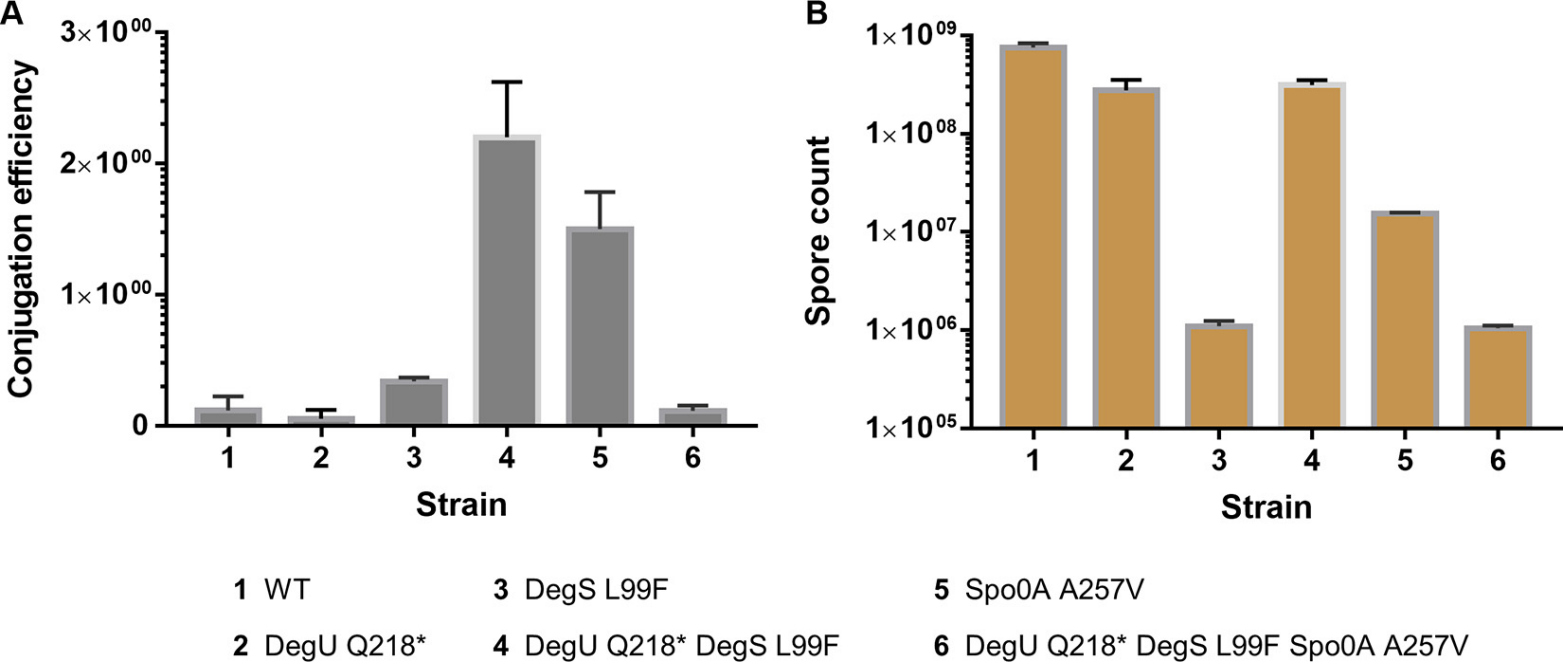
Evaluation of conjugation efficiency (A) and sporulation (B) of the wild-type (WT) and mutant strains of P. polymyxa. Spore count of the different variants was obtained from counting the spores of the samples from the fermenters by using phase-contrast microscopy using C-Chip disposable counting chambers. Error bars indicate the standard deviation.

Bacterial conjugation is one of the complex cellular functions which is influenced by different cellular and environmental conditions. In this study, we investigated the role of DegU, DegS, and Spo0A in the genetic accessibility of P. polymyxa. For this, we performed conjugation between P. polymyxa and Escherichia coli S17-1 harboring the nontargeting pCasPP plasmid without the homology-directed repair (HDR) template. Among the five engineered mutant strains, only DegU Q218* resulted in reduced conjugation efficiency, which was almost 70% in comparison to the wild-type. Meanwhile, DegS L99F and the triple mutant DegU Q218* DegS L99F Spo0A A257V showed slightly increased efficiencies. Remarkably, the double mutant DegU Q218* DegS L99F and single mutant Spo0A A257V possessed significantly improved conjugation efficiency, 19- and 13-fold higher than the wild-type, respectively. While the double mutant showed a substantial increase in the conjugation efficiency, the combined triple mutant of DegU Q218* DegS L99F Spo0A A257V reduced the efficiency toward the wild-type level (Fig. 4A). This indicates that conjugation is regulated via a different antagonistic mechanism in P. polymyxa involving the DegS/DegU and the Spo0A network.

It is interesting to observe that DegU, DegS, and Spo0A play a role in regulating the conjugation of P. polymyxa, as in B. subtilis transformation, these regulatory networks have been mainly described to control the development of natural competence (33). In B. subtilis, the master regulator of the natural competence, ComK, has been studied extensively. It was identified that the nonphosphorylated form of DegU binds to *comK* and thus increases the strain’s ability for plasmid uptake (33, 34). Furthermore, phosphorylated Spo0A (Spo0A~P) acts as an inhibitor of the natural competence repressor AbrB (35). On the other hand, conjugation is a complex process which is controlled by a multilayered regulation involving different regulatory proteins (36). Multiple factors could affect the efficiency of plasmid conjugation into B. subtilis, including the phospholipid composition of the cell membrane (37).

Meanwhile, no study has been reported so far on how genetic accessibility of P. polymyxa is regulated. Genome analysis revealed that P. polymyxa DSM 365 carries the *abrB* gene but is lacking *comK*, which could explain its inability for natural competence (38). In this study, it was found that the single mutation of Spo0A A257V significantly increased the conjugation efficiency of P. polymyxa. Among others, Spo0A has also been described to regulate *de novo* fatty acid and membrane lipid synthesis (39), which, in turn, might affect the conjugation process. Altogether, it can be evidenced that Spo0A has a pivotal role in controlling conjugation and thus might be a prominent target to optimize the genetic accessibility of P. polymyxa.

### Sporulation.

Following the late exponential phase or unfavorable environmental conditions, some bacteria may respond to the suboptimal environment by sporulation or biofilm formation. Sporulation allows the bacteria to survive in a dormant state and germinate again when the condition permits. Different genes are involved in sporulation, but it is well-known that Spo0A is the master regulator which regulates the early stage of sporulation. Activation of Spo0A is achieved through a phosphorylation cascade which includes a number of histidine kinases (4). In this study, we found that all the engineered strains can still form endospores, although the level of sporulation varies between the mutant strains (Fig. 4B). Compared to the mutants, the wild-type strain showed the highest level of sporulation. DegU Q218* and the double mutant DegU Q218* DegS L99F have slightly lower endospore numbers than the wild-type. On the contrary, the single mutant DegS L99F and the triple mutant DegU Q218* DegS L99F Spo0A A257V show 1,000-fold-lower endospore formation. To our surprise, the A257V mutation of the Spo0A does not abolish the endospore formation. Primarily found in B. subtilis, this mutation is known to cause a weakening of the promoter binding site by reduced homodimer formation or a decreased contact with the cognate sigma factors (11). Furthermore, Spo0A A257V was found to abolish sporulation via repression of either *spoIIA* or *spoIIG* while retaining the repression of the transition state regulator *abrB* on the same level (13). Analysis of the crystal structure of the B. subtilis Spo0A identified that the effect of A257V mutation was repressed by L174F and H162R. Therefore, in our case, we hypothesize that the alteration of L174 to Q174 in P. polymyxa in comparison to B. subtilis might suppress the effect of A257V mutation (Fig. S2), as observed in a sporulating B. anthracis strain (30). In addition, the effect of DegU~P on sporulation has been studied in B. subtilis in which DegU~P influences the level of Spo0A~P. A high level of DegU~P enhances sporulation by increasing the level of cellular Spo0A~P (40). This result leads us to hypothesize that DegU mutations investigated in this study might result in lower phosphorylation of DegU, which, in consequence, puts the Spo0A~P level below the threshold for sporulation initiation. This hypothesis is supported by the result of the plate-based assay in which the DegU Q218* mutation led to abolishment of the swarming motility and production of degradative enzymes (Fig. 3; Fig. S4).

### Bioreactor cultivation.

**FIG 5:**
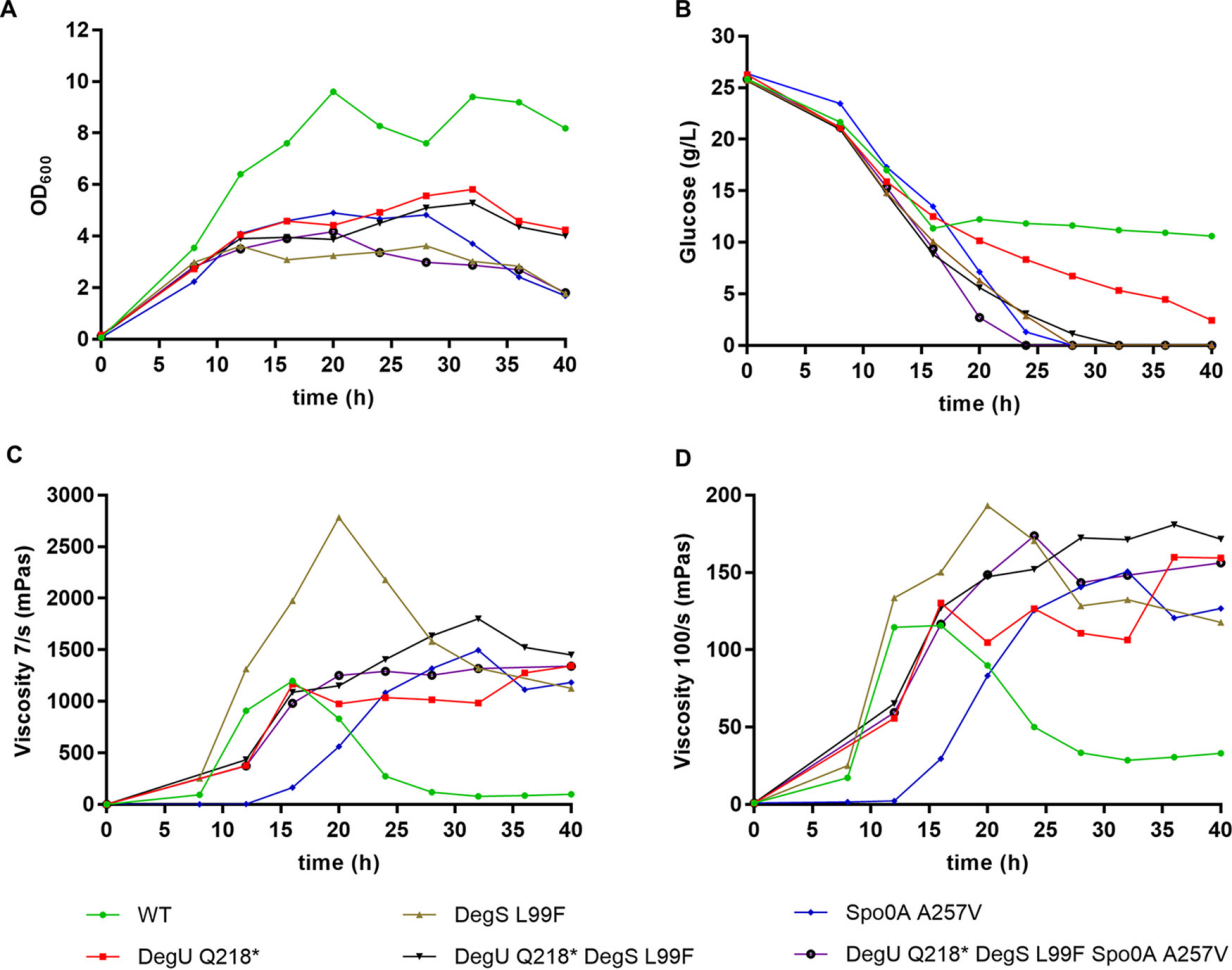
Characterization of the wild-type (WT) and engineered strains of P. polymyxa DSM 365 in 21 L-scale fermenters. (A) Growth profile, assessed via OD_600_ measurement. (B) Glucose consumption profile. (C and D) Viscosity profile at 7/s (C) and 100/s (D) shearing rates. The strain cultivations were performed as single experiments in 21-L bioreactors containing 12 L cultivation medium.

P. polymyxa is a highly promising EPS-producing bacterium which can produce both heteropolymers and homopolymers, depending on the carbon source. It produces heteropolymeric EPSs when excess glucose is available as the carbon source. When sucrose is available as the carbon source, its EPS biosynthesis shifts to the production of levan, which is a pure fructose polymer (14, 15, 41). Production of these EPSs will result in increased viscosity of the cultivation broth. In this study, cultivation in a glucose-based EPS-inducing medium revealed strong differences in the growth and viscosity profiles among the different P. polymyxa variants. All investigated strains produced a highly viscous culture broth, but the timing of viscosity formation, as well as the stability and the rheological properties, was different among the strains. Remarkably, all the engineered mutants showed significantly reduced biomass formation as measured by optical density at 600 nm (OD_600_) compared to the wild-type strain, even though the latter did use only half of the glucose provided as the carbon source (Fig. 5A and B). Based on rheological analyses with an applied shear stress of 7/s and 100/s, DegS L99F showed the highest viscosity (Fig. 5C and D), whereas mutants carrying a DegU Q218* mutation showed the highest viscosity at an elevated sheering rate of 1,000/s (Fig. S5). Moreover, the DegS L99F single mutant, distantly followed by the wild-type strain, showed the fastest viscosity formation within the first 20 h of the cultivation. However, both the wild-type and DegS L99F strains initiated a strong degradation of the viscosifying matrix after 16 to 20 h cultivation time, resulting in a significant decrease of viscosity in the subsequent course of cultivation. Interestingly, when incorporating a DegU Q218* mutation into the wild-type as well as in the DegS L99F mutant, the breakdown of the EPSs was mostly stopped, and thus, they switched toward a stable viscosity formation without further degradation. As a consequence, the final culture broth viscosities of DegU Q218* and DegU Q218* DegS L99F strains were higher than the wild-type and the DegS L99F single mutant. On the other hand, the Spo0A A257V single mutant showed a >12-h delay in viscosity formation despite having similar growth and cultivation profile to the DegU mutant. Again, in combination with the DegU Q218* mutation, viscosity formation of the Spo0A mutant was accelerated and finally reached a higher viscosity level.

## DISCUSSION

**FIG 6:**
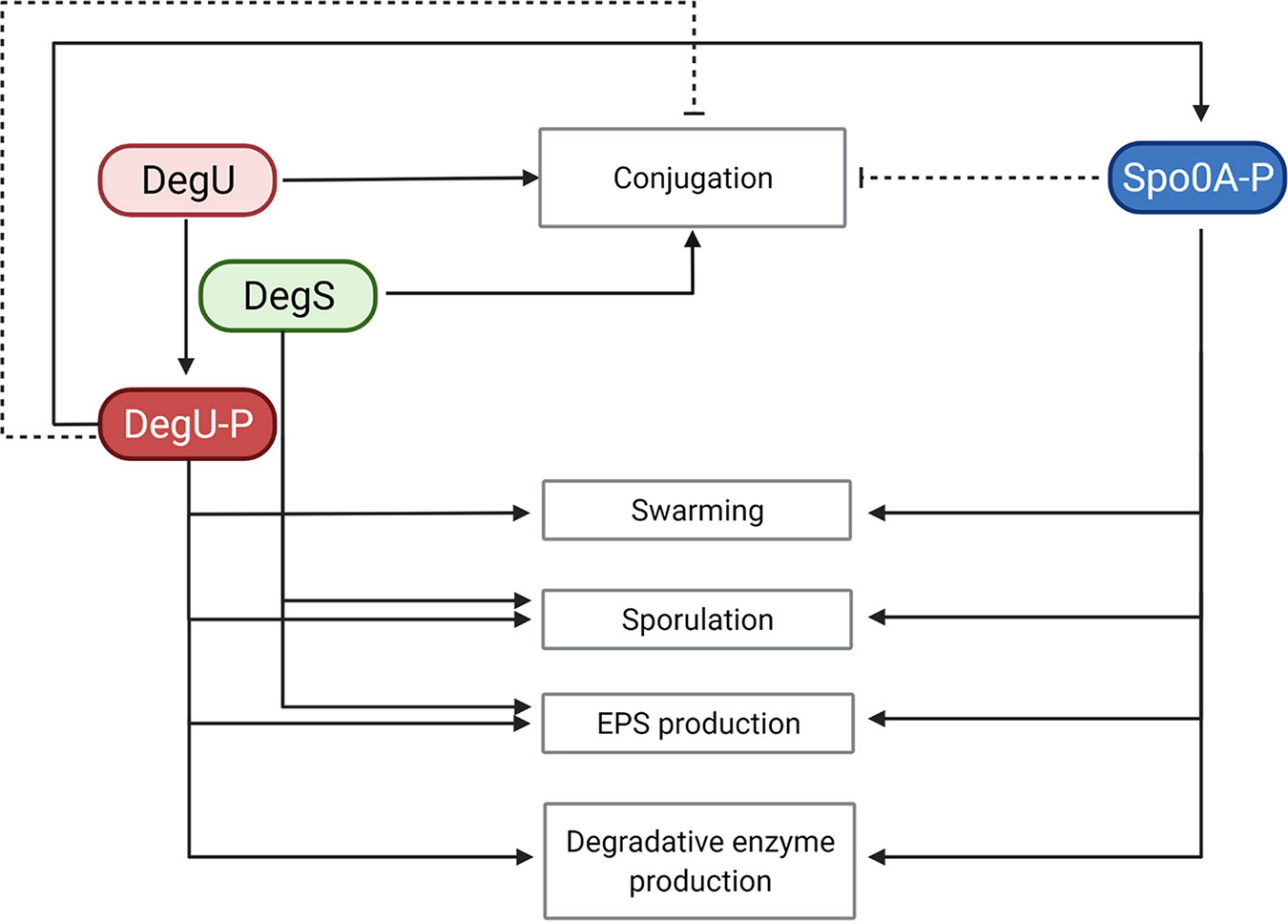
Proposed schematic model of regulatory network between DegU, DegS, and Spo0A in regulating complex cells behaviors in P. polymyxa DSM 365. Solid line indicates positive regulation, and dashed line indicates negative regulation. Phosphorylation of DegU is catalyzed by DegS. DegU~P positively regulates the activation of Spo0A, which, in turn, also regulates multiple cell behaviors.

In previous studies, we have developed efficient CRISPR-Cas-based systems to facilitate gene deletions and regulations in P. polymyxa DSM 365 (15, 18, 42, 43). Here, we demonstrated the reliability of the system to introduce targeted point mutations into the DegU, DegS, and Spo0A regulatory proteins, which are rarely investigated in P. polymyxa. Using the defined mutants generated by targeted integration of specific single nucleotide polymorphisms (SNPs) via CRISPR-Cas9, we propose the ability of DegU, DegS, and Spo0A in P. polymyxa to act as a phosphorelay-dependent multistage switch, which gradually controls various adaptive traits (Fig. 6). In many bacteria, these three proteins are known as pleiotropic response regulators which govern multiple cellular functions. Many of these functions are under regulation of phosphorylated DegU (DegU~P), including motility, biofilm formation, and degradative enzyme production. To date, natural competence development is the only known cellular property which is regulated by DegU in its nonphosphorylated form (33).

Depending on its gradual level of phosphorylation, DegU regulates multiple cellular functions by activating their transcription via binding to the promoter regions (2, 3, 44). DegU has different binding affinities for different promoter regions, and swarming motility is among the first cellular functions that are regulated by DegU~P (45). It was suggested that a low level of DegU~P is sufficient to activate flagellar biosynthesis, while hyperphosphorylation inhibits swarming motility in B. subtilis (32, 46, 47). In this study, it was found that the Q218* mutation in DegU protein led to abolishment of swarming motility. However, this phenomenon is unlikely to be caused by hyperphosphorylation of DegU, considering that the DegU Q218* mutant did not give a positive protease assay, as would be the case when hyperphosphorylation of DegU occurs (see Fig. S4 in the supplemental material) (1, 44, 48). Therefore, assuming a multilevel-switch activation of genes by DegU~P, one can speculate that DegU Q218* causes a medium level of DegU~P because swarming motility is repressed (as expected at low levels of DegU~P), while EPSs production is not altered (medium DegU~P), but protease expression is not present (as expected at high DegU~P).

In B. subtilis, Spo0A A257V was found to abolish sporulation while retaining the same repression level of the transition-state regulator *abrB* (13). Hence, it was not found to alter the development of natural competence. No further study has investigated whether the mutation also affects the conjugation efficiency in B. subtilis. In contrast, this particular mutation apparently did not abolish the spore formation in P. polymyxa. Interestingly, it had a great impact on the genetic accessibility of P. polymyxa, as observed from the increased conjugation efficiency. This result particularly highlights the differences in the regulatory functions of Spo0A in B. subtilis and P. polymyxa.

Characterization of the growth and viscosity profile in the fermenters provided insights into the EPS formation of the wild-type and the mutant strains of P. polymyxa. To our surprise, the wild-type and DegS L99F strains seem to undergo EPS degradation, as indicated by decreased viscosity of the culture broth over the course of the cultivation process. Interestingly, these two strains were the only ones which showed the swarming phenotype, while the other mutant strains did not. As swarming behavior and biofilm formation are reversely related (49), it is plausible that the wild-type and DegS L99F hydrolyze their own EPSs to enhance the swarming motility and to supply the nutrients to accommodate for increased flagellar synthesis.

As the heteropolymeric EPSs from P. polymyxa DSM 365 are composed of glucose, galactose, fucose, mannose, and glucuronic acid as the sugar monomers (15), its breakdown requires the release of hydrolytic glycosidases. The proteomics and lipidomics study of P. polymyxa showed increased abundance of glycoside hydrolases during swarming (31). Furthermore, transcriptomic analysis of an industrial P. polymyxa production strain carrying a mutation within the DNA binding domain of DegU indicated a strongly reduced expression of amylases, glucosidases, galactosidases, cellulases, levanases, as well as proteases, that may, in part, play a crucial role in the dismantling of the EPSs produced (unpublished data). These findings are consistent with our observations using skim milk agar plates for protease activity screening in which the DegU and Spo0A mutant strains showed a lack of lysis zones compared to the wild-type and the DegS L99F strains. Thus, a nonfunctional DegU regulator in P. polymyxa should lead to a higher, or even more stable, viscosity of the broth, especially at the later stage of cultivation, as demonstrated in Fig. 5C and D.

Unlike the DegU Q218* mutant, which resulted in decreased conjugation efficiency, the DegS L99F strain showed 3-fold-higher conjugation efficiency than the wild-type. From this, we conclude that the strain may have an altered phosphorelay of DegU toward lower levels of DegU~P with the consequence of higher EPSs production without reducing the production of degradative enzymes. Moreover, in our study, the DegU Q218* DegS L99F double mutant resulted in the highest viscosity formation and conjugation efficiency, suggesting that EPSs formation requires only minor levels of DegU~P.

Luo et al. (22) have proposed two EPS biosynthesis pathways in P. polymyxa based on comparative genome analysis with B. subtilis involving both regulatory systems. On the one hand, activation of Spo0A is mediated by autophoshorylation of *kinB* and subsequent stimulation of the *spo0F* response regulator transferring a phosphate group to Spo0A and thus activating biofilm formation. As discussed above, it has been shown that Spo0A in B. subtilis negatively regulates *abrB*, which is a negative regulator of genes involved in EPS formation (2). While Spo0A A257V was found to not affect repression of *abrB* expression in B. subtilis (13), it may partially repress *abrB* expression in P. polymyxa. As a result, EPS formation would only occur at the later stages of cultivation when Spo0A~P is present at higher intracellular levels. Consequently, the Spo0A A257V mutant was found to strongly delay EPS production during cultivation in the fermenter, while the triple mutant DegU Q218* DegS L99F Spo0A A257V showed similar early timing and levels of viscosity formation to the DegU Q218* DegS L99F double mutant. This indicates the presence of a second and alternative route for activation of the EPSs biosynthesis. Hence, the sensor histidine kinase DegS is triggered by an external stimulus followed by a subsequent phosphorelay of the response regulator DegU and thereby activates the biofilm genes. Since DegS/DegU promote viscosity formation through an alternative intracellular signaling pathway, their respective mutations cause strong EPS formation that seems to cover up the altered EPS formation through the Spo0A mutation in the same strain. From an evolutionary point of view, the regulation of EPS formation through separate independent regulators is beneficial, as biofilm formation can have different purposes, such as nutrient concentration or protection from harsh conditions (50).

### Conclusion.

In this study, different engineered strains were generated by the introduction of targeted point mutations facilitated by a CRISPR-Cas9-based system. The investigations of the pleiotropic regulators DegU, DegS, and Spo0A have provided insights into their roles in regulating different cellular responses and behaviors. In particular, some of the engineered mutants are found to be superior in comparison to the wild-type, particularly the double mutant DegU Q218* DegS L99F, with significantly improved conjugation efficiency. Furthermore, in contrast to the wild-type, this strain does not undergo reduced viscosity of the cultivation broth, which indicates stable EPS production without any further degradation. While some similarities are observed between P. polymyxa and B. subtilis, several physiological traits seem to be regulated differently. In particular, mutation in the Spo0A does not eliminate the sporulation, which is just the opposite of B. subtilis. Therefore, further investigations are needed to elucidate the roles and interactions of these regulatory proteins in coordinating complex cell behaviors as well as EPS production. Finally, based on the results obtained by this study, we propose a model of the roles of DegU, DegS, and Spo0A in regulating the conjugation, swarming motility, sporulation, EPS formation, and degradative enzyme production in P. polymyxa DSM 365.

## MATERIALS AND METHODS

### Strains and cultivation conditions.

P. polymyxa DSM 365 was obtained from the German Collection of Microorganisms and Cell Culture (DSMZ), Germany. Plasmid cloning and multiplication were performed in Escherichia coli DH5α (New England Biolabs, USA). E. coli S17-1 (ATCC 47055) was used as a conjugative donor strain to mediate the transformation of P. polymyxa. The strains were cultivated in LB media (10 g/L peptone, 5 g/L yeast extract, and 5 g/L NaCl). For plate media, an additional 1.5% of agar was used. Whenever necessary, the media were supplemented with 50 μg/mL neomycin and 20 μg/L polymyxin. P. polymyxa was cultivated at 30°C, while E. coli was cultivated at 37°C unless stated otherwise. For liquid culture, the strains were cultivated in 3 mL of LB media in 13-mL culture tubes and incubated at 250 rpm. The strains were stored as cryo-cultures in 24% glycerol and kept at −80°C for longer storage.

### Conjugation.

Conjugation was performed between P. polymyxa (recipient strain) and E. coli S17-1 harboring the plasmid of interest (donor strain). The cryo-cultures of both strains were streaked on LB plates containing, if necessary, suitable antibiotic. Subsequently, overnight liquid cultures were prepared from the colonies obtained on the plates. The overnight cultures were diluted 1:100 in 3 mL LB media, with or without antibiotic, in 13-mL plastic culture tubes. The cultures were cultivated at 37°C, 250 rpm, for 4 h. Subsequently, 900 μL of the recipient strain was heat shocked at 42°C for 15 min and mixed with 300 μL of the donor culture. The mixture was centrifuged at 6,000 × *g* for 3 min, and then the supernatant was discarded. The cell pellet was resuspended in 100 μL of LB media, and the resuspension was dropped on an LB agar plate. After overnight incubation at 30°C, the cells were scraped off the plate and resuspended in 150 μL of LB media. Afterward, the resuspension was plated on a selective LB agar plate containing 50 μg/mL neomycin and 20 μg/mL polymyxin, according to Rütering et al. (15). If necessary, the resuspension was diluted with appropriate dilution to obtain countable colonies on the plates. The plate was incubated at 30°C for 48 h to obtain P. polymyxa exconjugants. Screening of the exconjugants was performed by colony PCR and sequencing of the resulting DNA fragments. Plasmid curing was performed by 1:100 subcultivation of the strains every 24 h at 37°C.

### Plasmid construction.

Targeted point mutations were realized by a CRISPR-Cas9-mediated system by use of the pCasPP plasmid as a vector base. It represents a one-plasmid system which contains the Streptococcus pyogenes *cas9*-encoding gene (SpCas9) and the respective CRISPR-RNA (crRNA) under the control of the *sgsE* from Geobacillus stearothermophilus and *gapdh* promoter from Eggerthella lenta, respectively (15, 51). To realize Cas9 targeting the different positions in *degU*, *degS*, and *spo0A* genes, the most appropriate spacer sequences were chosen based on their closest proximity to the targeted regions. The selected spacer was located directly upstream of the NGG protospacer adjacent motif (PAM) site, which is recognized by the SpCas9. Approximately 1-kb homologous regions upstream and downstream of the targeted sites were amplified from the genomic DNA (gDNA) of P. polymyxa and provided as the repair template for homology-directed repair (HDR). Isolation of the gDNA was performed by using DNeasy blood and tissue kit (Qiagen), and purification of the PCR fragments was done using the Monarch gel purification kit (NEB). All fragments for plasmids cloning were amplified using Q5 DNA polymerase (NEB), and the plasmids were assembled by isothermal assembly. The list of strains and plasmids used in this study is provided in Table 1.

**TABLE 1:** List of strains and plasmids used in this study

Strain or plasmid	Genotype or description	Source or reference
Strains		
P. polymyxa DSM 365	Wild-type	DSMZ
P. polymyxa DegU Q218*	Q218* mutation in DegU	This study
P. polymyxa DegS L99F	L99F mutation in DegS	This study
P. polymyxa DegU Q218* DegS L99F	Q218* mutation in DegU and L99F mutation in DegS	This study
P. polymyxa Spo0A A257V	A257V mutation in Spo0A	This study
P. polymyxa DegU Q218* DegS L99F Spo0A A257V	Q218* mutation in DegU, L99F mutation in DegS, and A257V mutation in Spo0A	This study
E. coli DH5α	Cloning strain, *fhuA2* Δ*(argF-lacZ)*U169 *phoA glnV44* Φ80 Δ*(lacZ)*M15 *gyrA96 recA1 relA1 endA1 thi-1 hsdR17*	NEB
E. coli DH5α::pCasPP	Cloning strain for plasmid pCasPP	15
E. coli DH5α::pCasPP-degU SNP	Cloning strain for plasmid pCasPP degU-SNP	This study
E. coli DH5α:: pCasPP-degS SNP	Cloning strain for plasmid pCasPP degS-SNP	This study
E. coli DH5α::pCasPP-spo0A SNP	Cloning strain for plasmid pCasPP Spo0A-SNP	This study
E. coli S17-1	Conjugation strain, *recA pro hsdR* RP42Tc::Mu-Km::Tn*7* integrated into the chromosome	ATCC
E. coli S17-1::pCasPP	Conjugation strain for plasmid pCasPP	15
E. coli S17-1::pCasPP-degU SNP	Conjugation strain for plasmid pCasPP degU-SNP	This study
E. coli S17-1::pCasPP-degS SNP	Conjugation strain for plasmid pCasPP degS-SNP	This study
E. coli S17-1::pCasPP-spo0A SNP	Conjugation strain for plasmid pCasPP Spo0A-SNP	This study
Plasmids		
pCasPP	Vector plasmid harboring SpCas9 and nontargeting crRNA, Neo^r^^*a*^	15
pCasPP-degU SNP	Plasmid for introduction of C-to-T point mutation in *degU* gene at nucleotide position 652, Neo^r^	This study
pCasPP-degS SNP	Plasmid for introduction of C-to-T point mutation in *degS* gene at nucleotide position 293, Neo^r^	This study
pCasPP-spo0A SNP	Plasmid for introduction of C-to-T point mutation in *spo0A* gene at nucleotide position 770, Neo^r^	This study

a Neo^r^, neomycin resistance.

Desired point mutations were introduced by the primers used for amplification of the homologous regions. For *degS* and *spo0A*, several silent mutations were also introduced in the primers to improve the editing efficiency. E. coli DH5α was transformed with the isothermal assembly mixture using the heat shock method. Screening of the colonies was performed by colony PCR using GoTaq Polymerase (Promega). Subsequently, the plasmids were isolated by using GeneJet plasmid miniprep kit (Thermo Fisher Scientific) and sent for sequencing to confirm the correct assembly. Next, E. coli S17-1 was transformed with the correctly assembled plasmid, also by use of the heat shock method. Synthesis of oligonucleotides and sequencing analysis were done by Eurofins (Germany). *In silico* plasmid cloning was performed by the use of SnapGene version 5.1.5. The list of oligonucleotides used in this study is provided in Table 2.

**TABLE 2:** List of oligonucleotides used in this study

Plasmid	Primer name	Sequence (5′→3′)	Purpose
pCasPP-degU SNP	pCasPP_bb2_fw	CTGTTACAGGCATATTCATATCAATGTCG	Plasmid construction
	degU_sgRNA_rev	TTTTTTGCAAAATACTGCTGGCGTATCCCCTTTCAGATACTCGC	Plasmid construction
	degU_sgRNA_fw	CAGCAGTATTTTGCAAAAAAGTTTTAGAGCTAGAAATAGCAAGTTAAAATAAGGC	Plasmid construction
	degU_rev	CTGGAGCGAACCTGTTTCTTCCGGCGGGCTTGATGCG	Plasmid construction
	degU_US_fw	GAAGAAACAGGTTCGCTCCAG	Plasmid construction
	degU_US_rev	CAACCTCCATTTTTTACAAAATACTGCTGAC	Plasmid construction
	degU_DS_fw	GTCAGCAGTATTTTGTAAAAAATGGAGGTTG	Plasmid construction
	degU_DS_rev	CTCCTAATAACACCCACCTTTCGAC	Plasmid construction
	bb3_degU_fw	GTCGAAAGGTGGGTGTTATTAGGAGGGCCAGGAACCGTAAAAAGG	Plasmid construction
	pCasPP_bb3_rev	CGACATTGATATGAATATGCCTGTAACAG	Plasmid construction
	degU_check_fw	CTAGTGCTTAGGACGGAAATTGTG	Screening and sequencing of *degU* mutant
	degU_check_rev	GTTTCACACAGCTTTGTTCCCC	Screening and sequencing of *degU* mutant
	seq_degU_1	GGTAAGCTCATTCAGCAACTCC	Screening and sequencing of *degU* mutant
	seq_degU_2	GTACTGGGTTTCTGTATGAGCGG	Screening and sequencing of *degU* mutant
pCasPP-degS SNP	pCasPP_bb2_fw	CTGTTACAGGCATATTCATATCAATGTCG	Plasmid construction
	degS_sgRNA_rev	TCTCGCGGAAAATCATCACAGCGTATCCCCTTTCAGATACTC	Plasmid construction
	degS_sgRNA_fw	TGTGATGATTTTCCGCGAGAGTTTTAGAGCTAGAAATAGCAAGTTAAAATAAGGC	Plasmid construction
	degS_rev	ACTCTTATCCCCTCTGACATCCGGCGGGCTTGATGCG	Plasmid construction
	degS_US_fw	GATGTCAGAGGGGATAAGAGTC	Plasmid construction
	degS_US_rev	CGGAAGATCATGACATCGAATTGAAGCTG	Plasmid construction
	degS_DS_fw	CTTCAATTCGATGTCATGATCTTCCGCGAGAA	Plasmid construction
	degS_DS_rev	CGCATAGCCTTCATGAACCG	Plasmid construction
	bb3_degS_fw	CGGTTCATGAAGGCTATGCGGGCCAGGAACCGTAAAAAGG	Plasmid construction
	pCasPP_bb3_rev	CGACATTGATATGAATATGCCTGTAACAG	Plasmid construction
	degS_check_rev	CTACGATATGACAAATGGCAGTGG	Screening and sequencing of *degS* mutant
	seq_degS_1	GGAATGACTGTAAAATGGGAACAAG	Screening and sequencing of *degS* mutant
	seq_degS_2	CTGGAGCGAACCTGTTTCTTC	Screening and sequencing of *degS* mutant
pCasPP-spo0A SNP	pCasPP_bb2_fw	CTGTTACAGGCATATTCATATCAATGTCG	Plasmid construction
	spo0A_gRNA_rv	TGTTCTCAATTCTCAGCTTAGCGTATCCCCTTTCAGATACTC	Plasmid construction
	spo0A_gRNA_fw	TAAGCTGAGAATTGAGAACAGTTTTAGAGCTAGAAATAGCAAGTTAAAATAAGGC	Plasmid construction
	pCasPP_spo0A_rv	AATTATGAATTCCTTCTGTCGCGGCGGGCTTGATGCG	Plasmid construction
	spo0A_US_fw	CGACAGAAGGAATTCATAATTCGATGTC	Plasmid construction
	spo0A_US_rev	TTTGTTCTCAATCCTTAGTTTATCTACTACCATC	Plasmid construction
	spo0A_DS_fw	AAACTAAGGATTGAGAACAAAGTGTCCTGAAAG	Plasmid construction
	spo0A_DS_rev	CATGCCTGTCCTCTTCCAAAC	Plasmid construction
	bb3_spo0A_fw	CGTTTGGAAGAGGACAGGCATGGGCCAGGAACCGTAAAAAGG	Plasmid construction
	bb3_rev	CGACATTGATATGAATATGCCTGTAACAG	Plasmid construction
	spo0A_check_fw	GCTGTGACACATGTATTTGTGAATG	Screening and sequencing of *spo0A* mutant
	spo0A_check_rv	TCAAATGACTGTATGTCCTTAAAGCC	Screening and sequencing of *spo0A* mutant
	seq_spo0A	CTAACCGTGTTCGCCAATTAG	Screening and sequencing of *spo0A* mutant

### Sequence alignment and protein modeling.

Sequence alignment of the DegU, DegS, and Spo0A protein sequences of P. polymyxa DSM 365 and B. subtilis 168 was performed by using Clustal Omega (52). Protein modeling of P. polymyxa DegU, DegS, and Spo0A proteins was performed using RoseTTAFold (53). Visualization and analysis of the modeled proteins were done using PyMOL.

### Swarming assay.

Single colonies of the wild-type and mutant strains of P. polymyxa were inoculated into 3 mL of LB media and cultivated overnight in a shaking incubator at 30°C and 250 rpm. The following day, 10 μL of the culture was dropped to the center of an LB plate containing 0.4% agar. Subsequently, the plate was sealed with parafilm and incubated at room temperature for 48 h.

### Conjugation efficiency evaluation.

Conjugation efficiency of the different variants was evaluated by conjugating the cured strains with E. coli S17-1 harboring the pCasPP plasmid, following the protocol as described above. To obtain countable colonies, serial dilutions were performed before plating the conjugated strains onto LB plates containing neomycin and polymyxin. The conjugation efficiency was calculated as the total number of exconjugants per viable recipient cells.

### Bioreactor cultivation.

For characterization of growth properties and viscosity formation, the strains were tested in bioreactor cultivations. As preculture, the strains were cultivated in 100 mL of modified tryptic soy broth (TSB) medium (30 g/L TSB [Becton Dickinson], supplemented with 3 g/L yeast extract, 20.9 g/L MOPS [morpholinepropanesulfonic acid], and 10 g/L glucose) in 1-L baffled shake flasks. The preculture was grown at 33°C and 150 rpm for 24 h. Subsequently, the precultures were transferred (1% [vol/vol]) to 21-L bioreactors (Techfors; Infors) containing 12 L MM1 P100 medium adapted from Rütering et al. (15) (1.67 g/L KH_2_PO_4_, 1.33 g/L MgSO_4_ · 7H_2_O, 0.05 g/L CaCl_2_ · 2H_2_O, 5g/L peptone from soy, 30 g/L glucose monohydrate, 5 mg/L thiamine hydrochloride, 5 mg/L nicotinic acid, 0.2 mg/L riboflavin, 0.05 mg/L biotin, 1 mg/L calcium pantothenate, 5 mg/L pyridoxine hydrochloride, 0.05 mg/L cyanocobalamin, 0.05 mg/L lipoic acid, 13 mg/L MnSO_4_ · H_2_O, 4 mg/L ZnCl_2_, 4.6 mg/L CuSO_4_ · 5H_2_O, 2.8 mg/L Na_2_MoO_4_ · 2H_2_O, 15 mg/L Fe_2_(SO_4_)_3_ · H_2_O, and 0.4 g/L citric acid). Cultivation was performed at 30°C for 40 h; pH was set to 6.8 and adjusted with H_3_PO_4_ (25%) and NaOH (1 M). In the bioreactor, the target dissolved oxygen level was set at ≥30% in a stirrer gas flow cascade. To prevent sheering of the EPSs produced, agitation was limited to 300 to 600 rpm while using a stirrer setup consisting of two propellers and one Rushton turbine, in which the latter was placed near the agitator shaft. To maintain oxygen supply, aeration was performed at 5 to 30 L/min at 0.5-bar pressure. Struktol J673 was used as antifoam agent. Samples of the culture broth were taken every 4 h. Growth, sporulation, and viscosity were assessed from the whole culture broth sample, whereas sugar consumption was analyzed from the supernatant obtained after a 5-min centrifugation at 13,000 × *g*.

### Spore quantification.

Spore counts in fermentation samples were evaluated by phase-contrast microscopy using C-Chip disposable counting chambers (Neubauer/NanoEntek) according to the manufacturer’s manual. For accurate counting, fermentation samples were serially diluted with sterile 0.9% NaCl solution. Generation of dilution series and counting of spore titers were done in triplicates for each sampling point.

### Sugar profiling.

Glucose consumption over the course of the cultivation in bioreactors was measured by high-performance liquid chromatography (HPLC) equipped with a refractive index (RI) detector and Aminex HPX-87H 300- by 7.8-mm column (Bio-Rad) at 30°C, 0.5-mL/min eluent flow rate (5 mM H_2_SO_4_), and 30-min runtime. For analysis, 2-mL fermentation samples were centrifuged for 5 min at 13,000 × *g*, and then the supernatant was filtered through a 0.2-μm membrane before being used for the HPLC analysis.

### Rheological analysis.

Rheological analysis of culture broth viscosity was conducted every 4 h over the course of the cultivations using an Anton Paar MCR 302 rheometer with double-gap geometry (measuring cup, DG26.7-SS; temperature, 30°C; sample volume, 5 mL of whole culture broth). The samples were preconditioned in a preshear experiment at a constant shear rate of 10/s for 100 s. Ten data points were recorded every 10 s. After preconditioning, viscosity was measured as a function of the shear rate. Therefore, the shear rate was logarithmically increased from 1/s to 100/s while logging a total of 25 data points.
