# Supplementary material for: Insights in the Complex DegU, DegS, and Spo0A Regulation System of Paenibacillus polymyxa by CRISPR-Cas9-Based Targeted Point Mutations
Source: Appl Environ Microbiol. 2022 May 19;88(11):e00164-22. doi: 10.1128/aem.00164-22 (PMC9195935; doi:10.1128/aem.00164-22)
Supplement: Supplemental file 1 — Fig. S1 to S5 and Table S1. Download aem.00164-22-s0001.pdf, PDF file, 0.9 MB [file aem.00164-22-s0001.pdf]

Supporting Information

Insights in the complex DegU, DegS, Spo0A regulation system of *Paenibacillus polymyxa* by CRISPR-Cas9-based targeted point mutations

Meliawati Meliawati,[a,b] Tobias May,[c] Jeanette Eckerlin,[c] Daniel Heinrich,[c] Andrea Herold,[c] and Jochen Schmid[a,b,*]

[a]Institute of Molecular Microbiology and Biotechnology, University of Münster, Corrensstrasse 3, 48149 Münster, Germany
[b]Department of Biotechnology and Food Science, Norwegian University of Science and Technology, 7034 Trondheim, Norway
[c]BASF SE, Carl-Bosch-Strasse 38, 67056 Ludwigshafen am Rhein, Germany
[*]Corresponding author. Institute of Molecular Microbiology and Biotechnology, University of Münster, Corrensstrasse 3, 48149 Münster, Germany. Email: jochen.schmid@uni-muenster.de

Table of Contents

Figure S1. Sequencing confirmation of DegU Q218*, DegS L99F, and Spo0A A257V mutant strains 2

Figure S2. Sequence alignments of DegU, DegS, and Spo0A of *P. polymyxa* DSM 365 and *B. subtilis* 168 3

Figure S3. Protein modeling of Spo0A wild type and A257V mutant 4

Figure S4. Preliminary screening of DegU Q218* mutants on skim milk agar 5

Figure S5. Viscosity profile of the cultivation broth over the course of cultivation in the bioreactors, at sheering rate of 1000/s 6

Table S1. Sequences of plasmids generated in this study 7

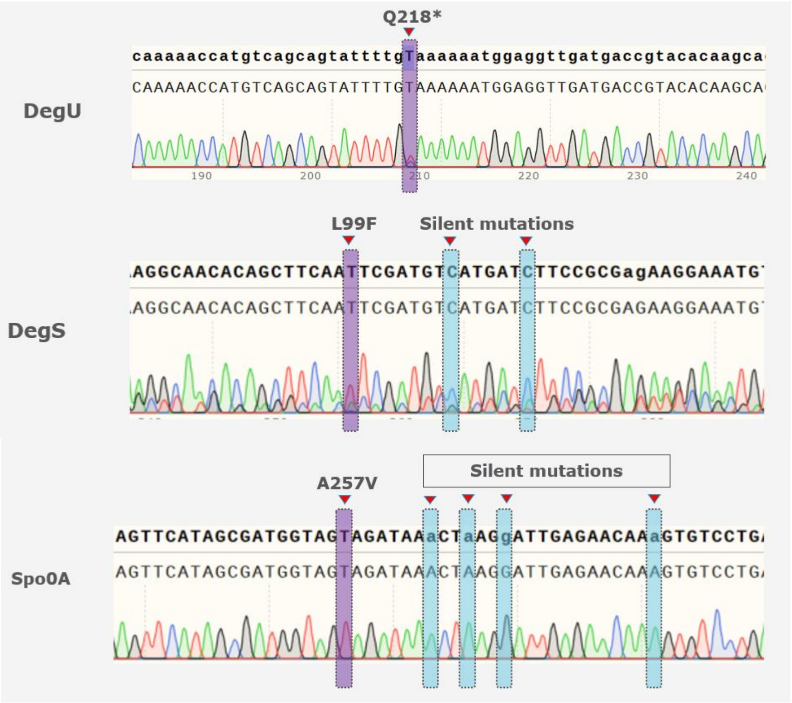

Figure S1. Sequencing confirmation of DegU Q218*, DegS L99F, and Spo0A A257V mutant strains. The targeted mutations are highlighted in purple, while the additional silent mutations are highlighted in blue.

A

```
P. polymyxa DSM 365     MENQEISNAPIKVLLADDHQLFREGLKRILNMEDDIEVIGECGDGIQVLEFCNVEKPDIV     60
B. subtilis 168         -------MTKVNIVIIDDHQLFREGVKRILDFEPTFEVVAEGDDGDEAARIVEHYHPDVV     53
                                : ::::: *********:****::*  :**:.* .** :. .: :  :**:*

P. polymyxa DSM 365     LMDINMPIENGVEATEKLREMFPDVKVIILSIHDDESYVFETLRKGANGYLLKDMEAESL     120
B. subtilis 168         IMDINMPNVNGVEATKQLVELYPESKVIILSIHDDENYVTHALKTGARGYLLKEMDADTL     113
                        :******  ******::* *::*: ***********.**  .:*:.**.*****:*:*::*

P. polymyxa DSM 365     INAIRSVHEGYAFIHPKVTGKLIQQLRRMTYLNETGAMAEGHTKEAGVKFVAGENNPLTR     180
B. subtilis 168         IEAVKVVAEGGSYLHPKVTHNLVNEFRRLATSGVSA-----HPQHEVYPEIRRPLHILTR     168
                        *:*:: * ** :::***** :*::::**::  . :.     * :.     :    : ***

P. polymyxa DSM 365     REAEVLRLMAEGKSNKMIGEYLFISEKTVKNHVSSILQKMEVDDRTQAVINSIKYGWVTL     240
B. subtilis 168         RECEVLQMLADGKSNRGIGESLFISEKTVKNHVSNILQKMNVNDRTQAVVVAIKNGWVEM     228
                        **.***:::*:****: *** *************.*****:*:******: :** *** :

P. polymyxa DSM 365     -   240
B. subtilis 168         R   229
```

B

```
P. polymyxa DSM 365     ---MDFQADIIDRVIKNAIQVMENSKYQMFEILDTARTELVTLNQELQSVLKETAETIEK     57
B. subtilis 168         MNKTKMDSKVLDSILMKMLKTVDGSKDEVFQIGEQSRQQYEQLVEELKQIKQQVYEVIEL     60
                            .:::.::* :: : ::.::.** ::*:* : :* :   * :**:.: ::. *.**

P. polymyxa DSM 365     VDQLEMNYRRSRIRLTEVSRDFVRYSEEDIKQAYEKATQLQLDVMIFREKEMYLKARRDD     117
B. subtilis 168         GDKLEVQTRHARNRLSEVSRNFHRFSEEEIRNAYEKAHKLQVELTMIQQREKQLRERRDD     120
                         *:**:: *::* **:****:* *:***:*::***** :**::: :::::*  *: ****

P. polymyxa DSM 365     LQKRAKSVEASVERAETIGSQMGVVLEYLSGELGQVTRIIESAKNRQFIGLKIILAQEEE     177
B. subtilis 168         LERRLLGLQEIIERSESLVSQITVVLNYLNQDLREVGLLLADAQAKQDFGLRIIEAQEEE     180
                        *::*  .::  :**:*:: **: ***:**. :* :*  :: .*: :* :**:** *****

P. polymyxa DSM 365     RKRISREIHDGPAQLLAHLVLRTEIVERMIAKQEFKMVQDEIVDLKKQVRSSLEEMRKVI     237
B. subtilis 168         RKRVSREIHDGPAQMLANVMMRSELIERIFRDRGAEDGFQEIKNLRQNVRNALYEVRRII     240
                        ***:**********:**::::*:*::**:: .:   :   :** :*:::**.:* *:*::*

P. polymyxa DSM 365     FNLRPMALDDLGLIPTLRKYVQDFEE---KTKIRSLFETRGKEHRLSSAMEAAIYRLIQE     294
B. subtilis 168         YDLRPMALDDLGLIPTLRKYLYTTEEYNGKVKIHFQCIGETEDQRLAPQFEVALFRLAQE     300
                        ::******************:   **   *.**:      . :::**:  :*.*::** **

P. polymyxa DSM 365     ALTNAAKHAYPTYVLVEITYQAQLVKIVVQDNGLGFKPELFQQKSKDHGHFGLIGMRERV     354
B. subtilis 168         AVSNALKHSESEEITVKVEITKDFVILMIKDNGKGFDLK--EAKEKKNKSFGLLGMKERV     358
                        *::** **:    : *::    ::* ::::*** **. :  : *.*.:  ***:**:***

P. polymyxa DSM 365     ELLEGRMEIESAENQGTKIVIHIPTNVEKGKE   386
B. subtilis 168         DLLEGTMTIDSKIGLGTFIMIKVPLSL-----   385
                        :**** * *:*  . ** *:*::* .:
```

C

```
P. polymyxa DSM 365     MQKIEVLLADDNREFTNLLAEYISDQEDMEVTGIAYNGEEVLQRIAESRNIPDVLILDII     60
B. subtilis 168         MEKIKVCVADDNRELVSLLSEYIEGQEDMEVIGVAYNGQECLSLFKE--KDPDVLVLDII     58
                        *:**:* :******:..**:***..****** *:****:* *. : *  : ****:****

P. polymyxa DSM 365     MPHLDGLGVLERLREMNLTPQPKIIMLTAFGQENITQRAVQLGASYYILKPFDMEVLANR     120
B. subtilis 168         MPHLDGLAVLERLRESDLKKQPNVIMLTAFGQEDVTKKAVDLGASYFILKPFDMENLVGH     118
                        *******.******* :*. **::*********::*::**:*****:******** *..:

P. polymyxa DSM 365     VRQLVGPQLV--SSSPVTISSMRSNVVPMGKTKNLDASITAIIHEIGVPAHIKGYQYLRE     178
B. subtilis 168         IRQVSGNASSVTHRAPSSQSSIIRSSQPEPKKKNLDASITSIIHEIGVPAHIKGYLYLRE     178
                        :**: *         :* : **:   .  *  *.********:************** ****

P. polymyxa DSM 365     AITMVYNNIEILGAITKTLYPAIAEKFKTTASRVERAIRHAIEVAWTRGNIDSISHLFGY     238
B. subtilis 168         AISMVYNDIELLGSITKVLYPDIAKKFNTTASRVERAIRHAIEVAWSRGNIDSISSLFGY     238
                        **:****:**:**:***.*** **:**:******************:******** ****

P. polymyxa DSM 365     TINISKSKPTNSEFIAMVADKLRIENKVS   267
B. subtilis 168         TVSMTKAKPTNSEFIAMVADKLRLEHKAS   267
                        *:.::*:*****************:*:*.*
```

Figure S2. Sequence alignments of DegU (A), DegS (B), and Spo0A (C) of *P. polymyxa* DSM 365 and *B. subtilis* 168. The targeted residues to be mutated in this study are highlighted in red.

A

B

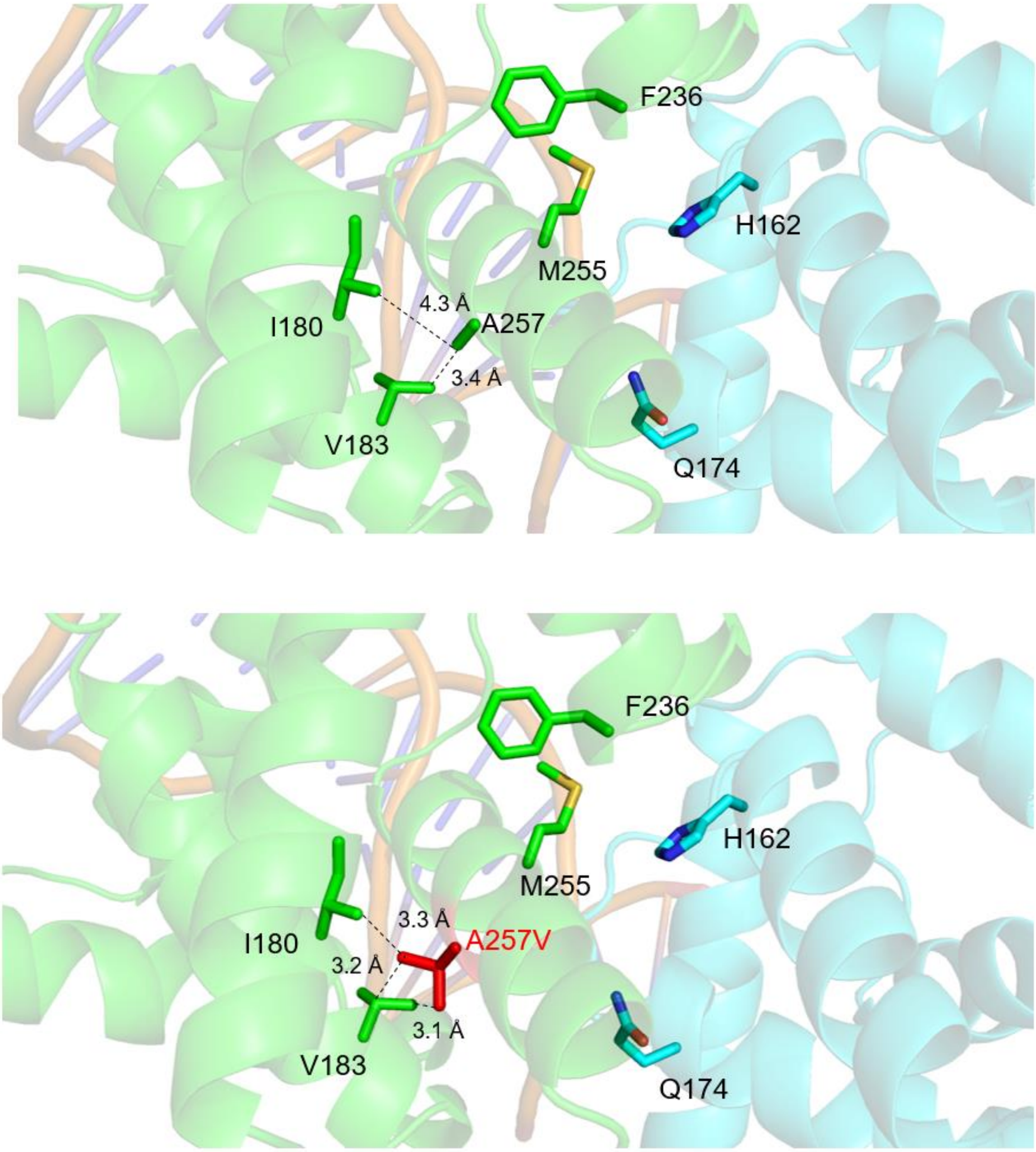

Figure S3. Protein modeling of Spo0A wild type (A) and A257V mutant (B). Main interactions between the amino acid at position 257 and nearby residues are highlighted. A257V mutation shorten the distance with the I180 and V183 residues, which might strengthen the interaction between the helices and thus weaken the interaction between the Spo0A dimer.

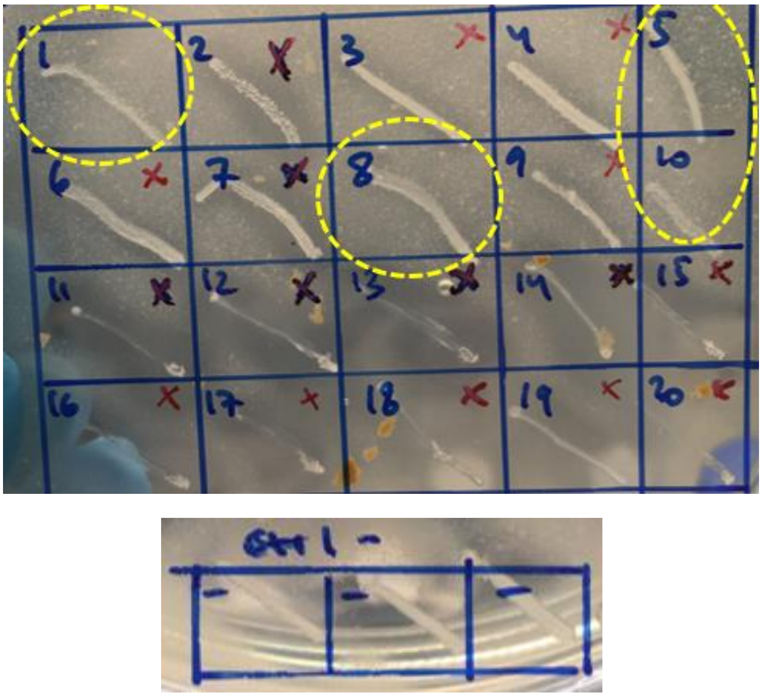

Figure S4. Preliminary screening of DegU Q218* mutants on skim milk agar. Colonies 1-20 were the screened exconjugants and the negative control (ctrl -) was the wild type strain. The growth of wild type resulted in a lysis zone which indicated the production of degradative enzymes. Meanwhile, the colonies harboring DegU Q218* mutation (highlighted in yellow) did not result in the lysis zone.

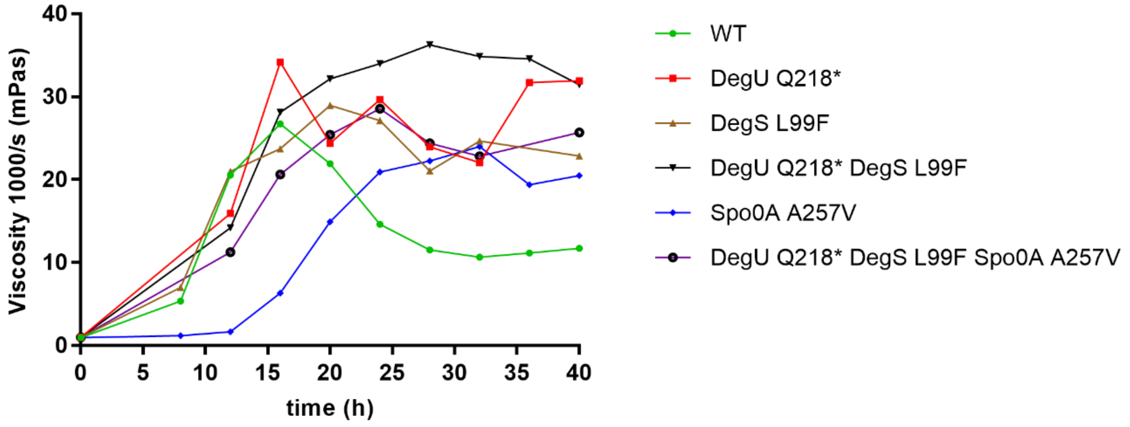

Figure S5. Viscosity profile of the cultivation broth over the course of cultivation in the bioreactors, at sheering rate of 1000/s. The strains cultivations were performed as single experiments in 21 L bioreactors containing 12 L cultivation medium.

Table S1. Sequences of plasmids generated in this study.

**pCasPP_degU-SNP**
ctgttacaggcatattcatatcaatgtcgccatcgatggaaataatttcaatggcatattctctaaaaaattt
gtatttagggctagaatttagaaagaaaatgtattattatagaagaggtagaacattaaggaatgatgtgaaa
gtgctagcactgaatgatagccttgctaataagtctatcatctggacacacaggatgatcttggttcatgtta
gcaccttttaggcaatgaatctactaaacggcagccgtttaggatcattgtcttaaagtttcataaaaacgaa
gggggattttaggcttttacttaatggacaagaagtacagcatcggcctggacatcggcaccaacagcgtggg
ctgggcggtcatcaccgacgagtacaaggtcccctccaagaagttcaaggtcctgggcaacaccgaccggcac
tcgatcaagaagaacctgatcggcgccctgctcttcgacagcggcgaaaccgccgaggcgacccgcctgaagc
ggaccgcccgtcgccgctacacccggcgcaagaaccgcatctgctacctgcaggagatcttctccaacgagat
ggccaaggtcgacgactcgttcttccaccggctcgaggagagcttcctggtggaggaggacaagaagcacgag
cgccacccgatcttcggcaacatcgtcgacgaggtggcctaccacgagaagtaccccaccatctaccacctcc
gcaagaagctggtcgactcgaccgacaaggcggacctgcggctcatctacctggccctcgcgcacatgatcaa
gttccgcggccacttcctcatcgagggcgacctgaacccggacaactccgacgtcgacaagctcttcatccag
ctggtgcagacctacaaccagctgttcgaggagaaccccatcaacgccagcggcgtcgacgccaaggcgatcc
tctccgcgcgcctgagcaagtcccggcgcctggagaacctcatcgcccagctgccgggcgagaagaagaacgg
cctcttcggcaacctgatcgcgctgtcgctcggcctgacccccaacttcaagagcaacttcgacctggccgag
gacgcgaagctccagctgtccaaggacacctacgacgacgacctggacaacctgctcgcccagatcggcgacc
agtacgcggacctcttcctggccgcgaagaacctctcggacgccatcctgctcagcgacatcctgcgggtcaa
caccgagatcaccaaggccccgctgtcggcgagcatgatcaagcggtacgacgagcaccaccaggacctgacc
ctgctcaaggccctcgtgcgccagcagctgcccgagaagtacaaggagatcttcttcgaccagtccaagaacg
gctacgccggctacatcgacggcggcgcgtcgcaggaggagttctacaagttcatcaagccgatcctggagaa
gatggacggcaccgaggagctgctcgtcaagctgaaccgcgaggacctgctccgcaagcagcggaccttcgac
aacggctccatcccgcaccagatccacctgggcgagctccacgccatcctccggcgccaggaggacttctacc
ccttcctgaaggacaaccgcgagaagatcgagaagatcctgaccttccggatcccgtactacgtcggcccccct
ggcccgcggcaactcccggttcgcgtggatgacccggaagtcggaggaaaccatcaccccgtggaacttcgag
gaggtcgtggacaagggcgcctccgcgcagtcgttcatcgagcgcatgaccaacttcgacaagaacctcccga
acgagaaggtcctgcccaagcacagcctgctctacgagtacttcaccgtgtacaacgagctgaccaaggtcaa
gtacgtgaccgagggcatgcggaagccggccttcctgtccggcgagcagaagaaggcgatcgtcgacctgctc
ttcaagaccaaccgcaaggtcaccgtgaagcagctgaaggaggactacttcaagaagatcgagtgcttcgact
ccgtcgagatctcgggcgtggaggaccgcttcaacgcctccctgggcacctaccacgacctgctcaagatcat
caaggacaaggacttcctcgacaacgaggagaacgaggacatcctggaggacatcgtcctcaccctgaccctc
ttcgaggaccgcgagatgatcgaggagcggctcaagacctacgcccacctgttcgacgacaaggtgatgaagc
agctgaagcggcgccggtacaccggctggggccgcctctcccggaagctgatcaacggcatccgggacaagca
gagcggcaagaccatcctggacttcctcaagtccgacggcttcgccaaccgcaacttcatgcagctcatccac
gacgactcgctgaccttcaaggaggacatccagaaggcccaggtgtccggccagggcgacagcctccacgagc
acatcgccaacctggcgggctccccggcgatcaagaagggcatcctccagaccgtcaaggtcgtggacgagct
ggtcaaggtgatgggccgccacaagcccgagaacatcgtgatcgagatggcccgggagaaccagaccacccag
aagggccagaagaactcccgcgagcggatgaagcgcatcgaggagggcatcaaggagctcggctcgcagatcc
tgaaggagcacccggtcgagaacacccagctccagaacgagaagctgtacctctactacctgcagaacggccg
cgacatgtacgtggaccaggagctcgacatcaaccggctgagcgactacgacgtcgaccacatcgtgccgcag
tccttcctgaaggacgactcgatcgacaacaaggtcctgacccgctccgacaagaaccggggcaagtccgaca
acgtgccctcggaggaggtcgtgaagaagatgaagaactactggcgccagctgctcaacgccaagctcatcac
ccagcgcaagttcgacaacctgaccaaggccgagcggggcggcctgtcggagctcgacaaggcgggcttcatc
aagcgccagctcgtcgaaacccggcagatcaccaagcacgtggcccagatcctggacagccggatgaacacca
agtacgacgagaacgacaagctgatccgcgaggtcaaggtgatcaccctcaagagcaagctggtgtccgactt
ccgcaaggacttccagttctacaaggtccgggagatcaacaactaccaccacgcccacgacgcgtacctgaac
gccgtcgtgggcaccgcgctgatcaagaagtacccgaagctggagtccgagttcgtctacggcgactacaagg
tctacgacgtgcgcaagatgatcgccaagtcggagcaggagatcggcaaggccaccgcgaagtacttcttcta
cagcaacatcatgaacttcttcaagaccgagatcaccctggccaacggcgagatccgcaagcggcccctgatc
gaaaccaacggcgaaaccggcgagatcgtctgggacaagggccgcgacttcgccaccgtccggaaggtgctgt
ccatgccgcaggtcaacatcgtcaagaaaaccgaggtgcagaccggcggcttcagcaaggagtccatcctccc
caagcgcaactcggacaagctgatcgcccggaagaaggactgggacccgaagaagtacggcggcttcgacagc
cccaccgtcgcctactccgtgctggtcgtggcgaaggtcgagaagggcaagagcaagaagctgaagtccgtga
aggagctgctcggcatcaccatcatggagcgctcctcgttcgagaagaacccgatcgacttcctggaggccaa
gggctacaaggaggtcaagaaggacctcatcatcaagctgcccaagtactcgctgttcgagctcgagaacggc
cgcaagcggatgctcgccagcgcgggcgagctgcagaagggcaacgagctggccctcccgtccaagtacgtca
acttcctgtacctcgcgtcccactacgagaagctgaagggctcgcccgaggacaacgagcagaagcagctctt
cgtggagcagcacaagcactacctggacgagatcatcgagcagatctcggagttcagcaagcgggtcatcctg

gccgacgcgaacctcgacaaggtgctgtccgcctacaacaagcaccgcgacaagccgatccgggagcaggcgg
agaacatcatccacctgttcaccctcaccaacctgggcgcccccgccgcgttcaagtacttcgacaccaccat
cgaccgcaagcggtacaccagcaccaaggaggtcctcgacgcgaccctgatccaccagtccatcaccggcctg
tacgaaacccgcatcgacctctcccagctcggcggcgactgagaattcagatctacgcgttccccgcaaaagc
ggcctttgactccctgcaagcctcagcgaccgaatatatcggttatgcgtgggcgatggttgttgtcattgtc
ggcgcaactatcggtatcaagctgtttaagaaattcacctcgaaagcaagctgataaaccgatacaattaaag
gctccttttggagcctttttttgctgctccttcggtcggacgtgcgtctacgggcaccttaccgcagccgtcg
gctgtgcgacacggacggatcgggcgaactggccgatgctgggagaagcgcgctgctgtacggcgcgcaccgg
gtgcggagcccctcggcgagcggtgtgaaacttctgtgaatggcctgttcggttgctttttttatacggctgc
cagataaggcttgcagcatctgggcggctaccgctatgatcggggcgttcctgcaattcttagtgcgagtatc
tgaaaggggatacgccagcagtattttgcaaaaaagttttagagctagaaatagcaagttaaaataaggctag
tccgttatcaacttgaaaaagtggcaccgagtcggtgctttttactccatctggatttgttcagaacgctcgg
ttgccgccgggcgttttttatctagctagacctgacttccgcctgcagggccagctcgcggacgtgctcatag
tccacgacgcccgtgattttgtagccctggccgacggccagcaggtaggccgacaggctcatgccggccgccg
ccgccttttcctcaatcgctcttcgttcgtctggaaggcagtacaccttgataggtgggctgcccttcctggt
tggcttggtttcatcagccatccgcttgccctcatctgttacgccggcggtagccggccagcctcgcagagca
ggattcccgttgagcaccgccaggtgcgaataagggacagtgaagaaggaacacccgctcgcgggtgggccta
cttcacctatcctgcccggctgacgccgttggatacaccaaggaaagtctacacgaaccctttggcaaaatcc
tgtatatcgtgcgaaaaaggatggatataccgaaaaaatcgctataatgaccccgaagcagggttatgcagcg
gaaaagatccgtcgacctgcatactagctgctcaaggtcgcccgcaaccggcgcatcaagcccgccggaagaa
acaggttcgctccagtcttgaggaaatgcgaaaggtcattttcaatttgcgtcctatggccctggatgacctg
ggacttattccgacgctccggaaatatgtgcaggattttgaagaaaaaacgaagattagatcgcttttttgaaa
caaggggcaaggaacaccgtctctcttccgcgatggaagcagccatttaccgtctgatccaggaagctttgac
caatgctgccaagcatgcctatcctacctatgtgcttgttgagattacctatcaggcgcagcttgtaaaaatc
gtggtgcaggataatggtttgggctttaagccagagctttttcagcaaaaaagcaaagatcatgggcattttg
gtctgattggtatgcgggaaagggttgaactgctcgaggggagaatggagatcgaatcagctgagaatcaagg
caccaagatagtgattcatatcccaaccaacgtggaaaagggaaaggagtaacaacatggaaaatcaagaaat
tagcaatgcacccattaaagttctcttagcggacgatcatcagttgttccgcgaaggacttaaacgtattttg
aatatggaggacgacattgaggtcatcggagaatgtggcgatggtattcaagtgttggaattctgtaacgtag
agaaaccggatattgttctgatggacattaatatgccaatcgaaaatggtgtagaggcaactgaaaaactgcg
tgagatgttcccggatgtcaaagttatcattttatccattcatgatgatgaaagctatgtatttgagacgttg
cgcaagggagccaatggttatctgttaaaggatatggaggccgagtccctcattaatgctattcgttcggttc
atgaaggctatgcgtttatccatccgaaggtaacaggtaagctcattcagcaactccgccggatgacctactt
gaatgaaacaggggctatggctgaaggtcatacgaaggaagccggcgtgaagtttgtcgcaggtgaaaataat
ccattgacccgtcgtgaagctgaagtattgcgtctgatggctgaaggtaagagcaacaagatgatcggtgaat
atttattcattagtgagaaaacggtcaaaaaccatgtcagcagtattttgtaaaaaatggaggttgatgaccg
tacacaagcagttattaactcaatcaaatatggatgggttacgctgtaaagtgtagtttttccatgcattaga
cgatgtagctgctaatagatatgaatgctgtaccgcctttcactccgggcgatatgtccgctcatacagaaac
ccagtacataaccgtgacacctcttcctgttatacggcatatattgtcgctataggaggaggtggaaccctcg
tgacggctcacttggtttggatattgcttatatacggtgtgggggccgctgtcgtgcatcttatgtatatacg
acaggcggttcaggcttatcacaatggcgaagattcatgcgcgatatcttcaggtcatcgcgcgattcgctat
attgtgattacgtccaatgacggagatcgtgtggaatgggtgatgcgttcgttgaacatacttgcctggctgc
accggaggccgctacatgtgacggtactggatgatgaatctatggacgatacgctaccaattctgacacggct
agtgcgctatagtcatatggatgtttcagtcatttcaagtcgtttttcaccggggaacccggttttaccgaag
tcaggcggtgaaaagcaggttgttattgatcttagaacagtcaagtcgttggcggacgccccgctggttcctt
agcgacaggggattgtggacatcgtggtcaaaatcggatataaatgtttataaacagttgtataggcaagaca
atagggatatcgttgagatctacttgatgtgaagcacacttcagggctattagccacagaagtgtgcttttt
gctgttctctggggtgataagaaccccaaaagtcatgaatgggaggagaaggggaaggtgaaaattgcagtat
atgcagtgaggtataaggggaattggaatctccgattatctgtggatattcgagtagatatgacatggtggat
gaatctagatgaggggcaggggaatgttgtcgaaaggtgggtgttattaggagggccaggaaccgtaaaaagg
ccgcgttgctggcgtttttccataggctccgcccccctgacgagcatcacaaaaatcgacgctcaagtcagag
gtggcgaaacccgacaggactataaagataccaggcgtttccccctggaagctccctcgtgcgctctcctgtt
ccgaccctgccgcttaccggatacctgtccgcctttctcccttcgggaagcgtggcgctttctcatagctcac
gctgtaggtatctcagttcggtgtaggtcgttcgctccaagctgggctgtgtgcacgaaccccccgttcagcc
cgaccgctgcgccttatccggtaactatcgtcttgagtccaacccggtaagacacgacttatcgccactggca
gcagccactggtaacaggattagcagagcgaggtatgtaggcggtgctacagagttcttgaagtggtggccta
actacggctacactagaagaacagtatttggtatctgcgctctgctgaagccagttaccttcggaaaaagagt
tggtagctcttgatccggcaaacaaaccaccgctggtagcggtggtttttttgtttgcaagcagcagattacg
cgcagaaaaaaaggatctcaagaagatcctttgatcttttctacggggtctgacgctcagtggaacgaaaact
cacgttaagggattttggtcatgagattatcaaaaaggatcttcacctagatccttttggttcatgtgcagct
ccatcagcaaaaggggatgataagtttatcaccaccgactatttgcaacagtgccgttaatggttataatagc

tgaataagaacggtgctctccaaatattcttatttagaaaagcaaatctaaaattatctgaaaagggaatgag
aatagtgaatggaccaataataatgactagagaagaaagaatgaagattgttcatgaaattaaggaacgaata
ttggataaatatggggatgatgttaaggctattggtgtttatggctctcttggtcgtcagactgatgggccct
attcggatattgagatgatgtgtgtcatgtcaacagaggaagcagagttcagccatgaatggacaaccggtga
gtggaaggtggaagtgaattttgatagcgaagagattctactagattatgcatctcaggtggaatcagattgg
ccgcttacacatggtcaatttttctctattttgccgatttatgattcaggtggatacttagagaaagtgtatc
aaactgctaaatcggtagaagcccaaacgttccacgatgcgatttgtgcccttatcgtagaagagctgtttga
atatgcaggcaaatggcgtaatattcgtgtgcaaggaccgacaacatttctaccatccttgactgtacaggta
gcaatggcaggtgccatgttgattggtctgcatcatcgcatctgttatacgacgagcgcttcggtcttaactg
aagcagttaagcaatcagatcttccttcaggttatgaccatctgtgccagttcgtaatgtctggtcaactttc
cgactctgagaaacttctggaatcgctagagaatttctggaatgggattcaggagtggacagaacgacacgga
tatatagtggatgtgtcaaaacgcataccatttgaacgatgacctctaataattgttaatcatgttggttac
gtatttattaacttctcctagtattagtaattatcatggctgtcatggcgcattaacggaataaaggtgtgc
ttaaatcgggccattttgcgtaataagaaaaaggattaattatgagcgaattgaattaataataaggtaatag
atttacattagaaaatgaaaggggattttatgcgtgagaatgttacagtctatcccggcattgccagtcgggg
atattaaaaagagtataggtttttattgcgataaactaggtttcactttggttcaccatgaagatggattcgc
agttctaatgtgtaatgaggttcggattcatctatgggaggcaagtgatgaagctggcgctctcgtagtaatg
attcaccggtttgtacaggtgcggagtcgtttattgctggtactgctagttgccgcattgaagtagagggaat
tgatgaattatatcaacatattaagcctttgggcattttgcaccccaatacatcattaaaagatcagtggtgg
gatgaacgagactttgcagtaattgatcccgacaacaatttgattagctttttcaacaataaaaagctaaa
atctattattaatctgttcagcaatcgggcgcgattgctgaataaaagatacgaaggtgatggttttgaactt
gttctttcttatcttgatacatatagaaataacgtcattttattttagttgctgaaaggtgcgttgaagtgt
tggtatgtatgtgttttaaagtattgaaaacccttaaaattggttgcacagaaaaaccccatctgttaaagtt
ataagtgactaaacaaataactaaatagatgggggtttcttttaatattatgtgtcctaatagtagcatttat
tcagatgaaaaatcaagggttttagtggacaagacaaaaagtggaaaagtgaggccatggagagaaaagaaaa
tcgctaatgttgattactttgaacttctgcatattcttgaatttaaaaaggctgaaagagtaaaagattgtgc
tgaaatattagagtataaacaaaatcgtgaaacaggcgaaagaaagttgtatcgagtgtggttttgtaaatcc
aggctttgtccaatgtgcaactggaggagagcaatgaaacatggcattcagtcacaaaaggttgttgctgaag
ttattaaacaaaagccaacagttcgttggttgtttctcacattaacagttaaaaatgtttatgatggcgaaga
attaaataagagtttgtcagatatggctcaaggatttcgccgaatgatgcaatataaaaaaattaataaaaat
cttgttggttttatgcgtgcaacggaagtgacaataaataataaagataattcttataatcagcacatgcatg
tattggtatgtgtggaaccaacttattttaagaatacagaaaactacgtgaatcaaaaacaatggattcaatt
ttggaaaaaggcaatgaaattagactatgatccaaatgtaaaagttcaaatgattcgaccgaaaaataaatat
aaatcggatatacaatcggcaattgacgaaactgcaaaatatcctgtaaaggatacggattttatgaccgatg
atgaagaaaagaatttgaaacgtttgtctgatttggaggaaggtttacaccgtaaaaggttaatctcctatgg
tggtttgttaaaagaaatacataaaaaattaaaccttgatgacacagaagaaggcgatttgattcatacagat
gatgacgaaaaagccgatgaagatggattttctattattgcaatgtggaattgggaacggaaaaattattta
ttaaagagtagttcaacaaacgggccagtttgttgaagattagatgctataattgttattaaaaggattgaag
gatgggag

pCasPP_degS-SNP

ctgttacaggcatattcatatcaatgtcgccatcgatggaaataatttcaatggcatattctctaaaaaattt
gtatttagggctagaatttagaaagaaaatgtattattatagaagaggtagaacattaaggaatgatgtgaaa
gtgctagcactgaatgatagccttgctaataagtctatcatctggacacacaggatgatcttggttcatgtta
gcacctttaggcaatgaatctactaaacggcagccgtttaggatcattgtcttaaagtttcataaaaacgaa
gggggattttaggcttttacttaatggacaagaagtacagcatcggcctggacatcggcaccaacagcgtggg
ctgggcggtcatcaccgacgagtacaaggtcccctccaagaagttcaaggtcctgggcaacaccgaccggcac
tcgatcaagaagaacctgatcggcgccctgctcttcgacagcggcgaaaccgccgaggcgacccgcctgaagc
ggaccgcccgtcgccgctacacccggcgcaagaaccgcatctgctacctgcaggagatcttctccaacgagat
ggccaaggtcgacgactcgttcttccaccggctcgaggagagcttcctggtggaggaggacaagaagcacgag
cgccacccgatcttcggcaacatcgtcgacgaggtggcctaccacgagaagtaccccaccatctaccacctcc
gcaagaagctggtcgactcgaccgacaaggcggacctgcggctcatctacctggccctcgcgcacatgatcaa
gttccgcggccacttcctcatcgagggcgacctgaacccggacaactccgacgtcgacaagctcttcatccag
ctggtgcagacctacaaccagctgttcgaggagaaccccatcaacgccagcggcgtcgacgccaaggcgatcc
tctccgcgcgcctgagcaagtcccggcgcctggagaacctcatcgcccagctgccgggcgagaagaagaacgg
cctcttcggcaacctgatcgcgctgtcgctcggcctgacccccaacttcaagagcaacttcgacctggccgag
gacgcgaagctccagctgtccaaggacacctacgacgacgacctggacaacctgctcgcccagatcggcgacc
agtacgcggacctcttcctggccgcgaagaacctctcggacgccatcctgctcagcgacatcctgcgggtcaa
caccgagatcaccaaggccccgctgtcggcgagcatgatcaagcggtacgacgagcaccaccaggacctgacc
ctgctcaaggccctcgtgcgccagcagctgcccgagaagtacaaggagatcttcttcgaccagtccaagaacg
gctacgccggctacatcgacggcggcgcgtcgcaggaggagttctacaagttcatcaagccgatcctggagaa

```
gatggacggcaccgaggagctgctcgtcaagctgaaccgcgaggacctgctccgcaagcagcggaccttcgac
aacggctccatcccgcaccagatccacctgggcgagctccacgccatcctccggcgccaggaggacttctacc
ccttcctgaaggacaaccgcgagaagatcgagaagatcctgaccttccggatcccgtactacgtcggcccct
ggcccgcggcaactcccggttcgcgtggatgacccggaagtcggaggaaaccatcaccccgtggaacttcgag
gaggtcgtggacaagggcgcctccgcgcagtcgttcatcgagcgcatgaccaacttcgacaagaacctcccga
acgagaaggtcctgcccaagcacagcctgctctacgagtacttcaccgtgtacaacgagctgaccaaggtcaa
gtacgtgaccgagggcatgcggaagccggccttcctgtccggcgagcagaagaaggcgatcgtcgacctgctc
ttcaagaccaaccgcaaggtcaccgtgaagcagctgaaggaggactacttcaagaagatcgagtgcttcgact
ccgtcgagatctcgggcgtggaggaccgcttcaacgcctccctgggcacctaccacgacctgctcaagatcat
caaggacaaggacttcctcgacaacgaggagaacgaggacatcctggaggacatcgtcctcaccctgaccctc
ttcgaggaccgcgagatgatcgaggagcggctcaagacctacgcccacctgttcgacgacaaggtgatgaagc
agctgaagcggcgccggtacaccggctggggccgcctctcccggaagctgatcaacggcatccgggacaagca
gagcggcaagaccatcctggacttcctcaagtccgacggcttcgccaaccgcaacttcatgcagctcatccac
gacgactcgctgaccttcaaggaggacatccagaaggcccaggtgtccggccagggcgacagcctccacgagc
acatcgccaacctggcgggctccccggcgatcaagaagggcatcctccagaccgtcaaggtcgtggacgagct
ggtcaaggtgatgggccgccacaagcccgagaacatcgtgatcgagatggcccgggagaaccagaccacccag
aagggccagaagaactcccgcgagcggatgaagcgcatcgaggagggcatcaaggagctcggctcgcagatcc
tgaaggagcacccggtcgagaacacccagctccagaacgagaagctgtacctctactacctgcagaacggccg
cgacatgtacgtggaccaggagctcgacatcaaccggctgagcgactacgacgtcgaccacatcgtgccgcag
tccttcctgaaggacgactcgatcgacaacaaggtcctgacccgctccgacaagaaccgggcaagtccgaca
acgtgccctcggaggaggtcgtgaagaagatgaagaactactggcgccagctgctcaacgccaagctcatcac
ccagcgcaagttcgacaacctgaccaaggccgagcggggcggcctgtcggagctcgacaaggcgggcttcatc
aagcgccagctcgtcgaaacccggcagatcaccaagcacgtggcccagatcctggacagccggatgaacacca
agtacgacgagaacgacaagctgatccgcgaggtcaaggtgatcaccctcaagagcaagctggtgtccgactt
ccgcaaggacttccagttctacaaggtccgggagatcaacaactaccaccacgcccacgacgcgtacctgaac
gccgtcgtgggcaccgcgctgatcaagaagtacccgaagctggagtccgagttcgtctacggcgactacaagg
tctacgacgtgcgcaagatgatcgccaagtcggagcaggagatcggcaaggccaccgcgaagtacttcttcta
cagcaacatcatgaacttcttcaagaccgagatcaccctggccaacggcgagatccgcaagcggcccctgatc
gaaaccaacggcgaaaccggcgagatcgtctgggacaagggccgcgacttcgccaccgtccggaaggtgctgt
ccatgccgcaggtcaacatcgtcaagaaaaccgaggtgcagaccggcggcttcagcaaggagtccatcctccc
caagcgcaactcggacaagctgatcgcccggaagaaggactgggacccgaagaagtacggcggcttcgacagc
cccaccgtcgcctactccgtgctggtcgtggcgaaggtcgagaagggcaagagcaagaagctgaagtccgtga
aggagctgctcggcatcaccatcatggagcgctcctcgttcgagaagaacccgatcgacttcctggaggccaa
gggctacaaggaggtcaagaaggacctcatcatcaagctgcccaagtactcgctgttcgagctcgagaacggc
cgcaagcggatgctcgccagcgcgggcgagctgcagaagggcaacgagctggccctcccgtccaagtacgtca
acttcctgtacctcgcgtcccactacgagaagctgaagggctcgcccgaggacaacgagcagaagcagctctt
cgtggagcagcacaagcactacctggacgagatcatcgagcagatctcggagttcagcaagcgggtcatcctg
gccgacgcgaacctcgacaaggtgctgtccgcctacaacaagcaccgcgacaagccgatccgggagcaggcgg
agaacatcatccacctgttcaccctcaccaacctgggcgcccccgccgcgttcaagtacttcgacaccaccat
cgaccgcaagcggtacaccagcaccaaggaggtcctcgacgcgaccctgatccaccagtccatcaccggcctg
tacgaaacccgcatcgacctctcccagctcggcggcgactgagaattcagatctacgcgttccccgcaaaagc
ggcctttgactccctgcaagcctcagcgaccgaatatatcggttatgcgtgggcgatggttgttgtcattgtc
ggcgcaactatcggtatcaagctgtttaagaaattcacctcgaaagcaagctgataaaccgatacaattaaag
gctcctttggagcctttttttgctgctccttcggtcggacgtgcgtctacgggcaccttaccgcagccgtcg
gctgtgcgacacggacggatcgggcgaactggccgatgctgggagaagcgcgctgctgtacggcgcgcaccgg
gtgcggagcccctcggcgagcggtgtgaaacttctgtgaatggcctgttcggttgctttttttatacggctgc
cagataaggcttgcagcatctgggcggctaccgctatgatcggggcgttcctgcaattcttagtgcgagtatc
tgaaaggggatacgctgtgatgattttccgcgagagttttagagctagaaatagcaagttaaaataaggctag
tccgttatcaacttgaaaaagtggcaccgagtcggtgctttttactccatctggatttgttcagaacgctcgg
ttgccgccgggcgtttttttatctagctagacctgacttccgcctgcagggccagctcgcggacgtgctcatag
tccacgacgcccgtgattttgtagccctggccgacggccagcaggtaggccgacaggctcatgccggccgccg
ccgcctttttcctcaatcgctcttcgttcgtctggaaggcagtacaccttgataggtgggctgcccttcctggt
tggcttggtttcatcagccatccgcttgccctcatctgttacgccggcggtagccggccagcctcgcagagca
ggattcccgttgagcaccgccaggtgcgaataagggacagtgaagaaggaacacccgctcgcgggtgggccta
cttcacctatcctgcccggctgacgccgttggatacaccaaggaaagtctacacgaaccctttggcaaaatcc
tgtatatcgtgcgaaaaaggatggatataccgaaaaaatcgctataatgaccccgaagcagggttatgcagcg
gaaaagatccgtcgacctgcatactagctgctcaaggtcgcccgcaaccggcgcatcaagcccgccggatgtc
agaggggataagagtctgaactggctgcgtgcggagctagaggatgcccaaggcaaaacagcatatattgatc
tcgctaaagtgatcgactggaacggttggaaaaccattaatgtagatatgagcggtttgaatatttcgtatcc
agcgaagctgaaacgtctttatgtggtcaatgtggcggaaggtcaggacgaacgtgctaaaacaggcaatgtt
gcttttgataatatttcttttaatacatcgggaacggtcggcacagaaggacttaaaaagggaacagctcaaa
```

```
tgacgatcggacaaaaatcaatgacagtaaacggaacacctacaacaattgatgcagctccgatgactcgcga
tggttcaacgtatgttccgattaagtatgtgctggacgcatttggtggacaagcaaaatggaatgcgggcgat
caacggatcaccgttatgcgcggtggtgtgctgatggatttgaccgtagggaaaaaagaagttgttctgaacg
ggaagcgtcaaagtactgatgttgcacccgttgtgctagggggtaggactttagtcccgttgagactcgtgtc
tgagcagttaggaatgactgtaaaatgggaacaagaaacgaagaccatcacccttcaatcatgatatggtatg
atatatacttgaaatgctaggattggagtagaacatttgtggactttcaagccgatatcatagaccgagtcat
taaaaatgccatccaggtgatggagaacagcaaatatcagatgttcgaaatattggacacggcccggaccgag
ctggtcacgttaaatcaggagctccagagcgtcttgaaggaaacagcagaaacgatcgaaaaggtggaccagc
tggaaatgaactatcggcggtcccgcattcggttgactgaggtcagccgtgacttcgttcgctattcggaaga
ggatatcaagcaggcttatgagaaggcaacacagcttcaattcgatgtcatgatcttccgcgagaaggaaatg
tatctcaaagccagaagagatgatcttcaaaagcgggccaaaagtgttgaggcttctgtcgagcgcgccgaaa
ccatcggttcgcagatgggagtcgtgctggaatatttgtcgggcgaattgggacaagtgacgcggatcatcga
atcggctaaaaaccggcagtttattggtctgaaaattattttggctcaggaagaggagcgcaaacgcatatcc
cgtgaaattcacgatggacctgcgcagctccttgcgcatctagtgcttaggacggaaattgtggaaagaatga
tcgccaagcaggaatttaagatggttcaggacgaaatagtagacttgaagaaacaggttcgctccagtcttga
ggaaatgcgaaaggtcattttcaatttgcgtcctatggccctggatgacctgggacttattccgacgctccgg
aaatatgtgcaggattttgaagaaaaaacgaagattagatcgctttttgaaacaaggggcaaggaacaccgtc
tctcttccgcgatggaagcagccatttaccgtctgatccaggaagctttgaccaatgctgccaagcatgccta
tcctacctatgtgcttgttgagattacctatcaggcgcagcttgtaaaaatcgtggtgcaggataatggtttg
ggctttaagccagagcttttcagcaaaaaagcaaagatcatgggcattttggtctgattggtatgcgggaaa
gggttgaactgctcgagggagaatggagatcgaatcagctgagaatcaaggcaccaagatagtgattcatat
cccaaccaacgtggaaaagggaaaggagtaacaacatggaaaatcaagaaattagcaatgcacccattaaagt
tctcttagcggacgatcatcagttgttccgcgaaggacttaaacgtattttgaatatggaggacgacattgag
gtcatcggagaatgtggcgatggtattcaagtgttggaattctgtaacgtagagaaaccggatattgttctga
tggacattaatatgccaatcgaaaatggtgtagaggcaactgaaaaactgcgtgagatgttcccggatgtcaa
agttatcattttatccattcatgatgatgaaagctatgtatttgagacgttgcgcaagggagccaatggttat
ctgttaaaggatatggaggccgagtccctcattaatgctattcgttcggttcatgaaggctatgcgggccagg
aaccgtaaaaaggccgcgttgctggcgtttttccataggctccgcccccctgacgagcatcacaaaaatcgac
gctcaagtcagaggtggcgaaacccgacaggactataaagataccaggcgtttccccctggaagctccctcgt
gcgctctcctgttccgaccctgccgcttaccggatacctgtccgcctttctcccttcgggaagcgtggcgctt
tctcatagctcacgctgtaggtatctcagttcggtgtaggtcgttcgctccaagctgggctgtgtgcacgacc
ccccgttcagcccgaccgctgcgccttatccggtaactatcgtcttgagtccaacccggtaagacacgactt
atcgccactggcagcagccactggtaacaggattagcagagcgaggtatgtaggcggtgctacagagttcttg
aagtggtggcctaactacggctacactagaagaacagtatttggtatctgcgctctgctgaagccagttacct
tcggaaaaagagttggtagctcttgatccggcaaacaaaccaccgctggtagcggtggtttttttgtttgcaa
gcagcagattacgcgcagaaaaaaaggatctcaagaagatcctttgatcttttctacggggtctgacgctcag
tggaacgaaaactcacgttaagggattttggtcatgagattatcaaaaaggatcttcacctagatccttttgg
ttcatgtgcagctccatcagcaaaaggggatgataagtttatcaccaccgactatttgcaacagtgccgttaa
tggttataatagctgaataagaacggtgctctccaaatattcttatttagaaaagcaaatctaaaattatctg
aaaagggaatgagaatagtgaatggaccaataataatgactagagaagaaagaatgaagattgttcatgaaat
taaggaacgaatattggataaatatggggatgatgttaaggctattggtgtttatggctctcttggtcgtcag
actgatgggccctattcggatattgagatgatgtgtgtcatgtcaacagaggaagcagagttcagccatgaat
ggacaaccggtgagtggaaggtggaagtgaattttgatagcgaagagattctactagattatgcatctcaggt
ggaatcagattggccgcttacacatggtcaatttttctctattttgccgatttatgattcaggtggatactta
gagaaagtgtatcaaactgctaaatcggtagaagcccaaacgttccacgatgcgatttgtgcccttatcgtag
aagagctgtttgaatatgcaggcaaatggcgtaatattcgtgtgcaaggaccgacaacatttctaccatcctt
gactgtacaggtagcaatggcaggtgccatgttgattggtctgcatcatcgcatctgttatacgacgagcgct
tcggtcttaactgaagcagttaagcaatcagatcttccttcaggttatgaccatctgtgccagttcgtaatgt
ctggtcaactttccgactctgagaaacttctggaatcgctagagaatttctggaatgggattcaggagtggac
agaacgacacggatatatagtggatgtgtcaaaacgcataccattttgaacgatgacctctaataattgttaa
tcatgttggttacgtatttattaacttctcctagtattagtaattatcatggctgtcatggcgcattaacgga
ataaagggtgtgcttaaatcgggccattttgcgtaataagaaaaaggattaattatgagcgaattgaattaat
aataaggtaatagatttacattagaaaatgaaaggggattttatgcgtgagaatgttacagtctatcccggca
ttgccagtcggggatattaaaaagagtataggttttattgcgataaactaggtttcactttggttcaccatg
aagatggattcgcagttctaatgtgtaatgaggttcggattcatctatgggaggcaagtgatgaagctggcgc
tctcgtagtaatgattcaccggtttgtacaggtgcggagtcgtttattgctggtactgctagttgccgcattg
aagtagagggaattgatgaattatatcaacatattaagcctttgggcattttgcaccccaatacatcattaaa
agatcagtggtgggatgaacgagactttgcagtaattgatcccgacaacaatttgattagctttttcaacaa
ataaaaagctaaaatctattattaatctgttcagcaatcgggcgcgattgctgaataaaagatacgaaggtga
tggttttgaacttgttctttcttatcttgatacatatagaaataacgtcattttatttagttgctgaaagg
tgcgttgaagtgttggtatgtatgtgttttaaagtattgaaacccttaaattggttgcacagaaaaacccc
```

atctgttaaagttataagtgactaaacaaataactaaatagatgggggtttcttttaatattatgtgtcctaa
tagtagcatttattcagatgaaaaatcaagggttttagtggacaagacaaaaagtggaaaagtgaggccatgg
agagaaaagaaaatcgctaatgttgattactttgaacttctgcatattcttgaatttaaaaaggctgaaagag
taaaagattgtgctgaaatattagagtataaacaaaatcgtgaaacaggcgaaagaaagttgtatcgagtgtg
gttttgtaaatccaggctttgtccaatgtgcaactggaggagagcaatgaaacatggcattcagtcacaaaag
gttgttgctgaagttattaaacaaaagccaacagttcgttggttgtttctcacattaacagttaaaaatgttt
atgatggcgaagaattaaataagagtttgtcagatatggctcaaggatttcgccgaatgatgcaatataaaaa
aattaataaaaatcttgttggttttatgcgtgcaacggaagtgacaataaataataaagataattcttataat
cagcacatgcatgtattggtatgtgtggaaccaacttattttaagaatacagaaaactacgtgaatcaaaaac
aatggattcaattttggaaaaaggcaatgaaattagactatgatccaaatgtaaaagttcaaatgattcgacc
gaaaaataaatataaatcggatatacaatcggcaattgacgaaactgcaaaatatcctgtaaaggatacggat
tttatgaccgatgatgaagaaaagaatttgaaacgtttgtctgatttggaggaaggtttacaccgtaaaaggt
taatctcctatggtggtttgttaaaagaaatacataaaaaattaaaccttgatgacacagaagaaggcgattt
gattcatacagatgatgacgaaaaagccgatgaagatggattttctattattgcaatgtggaattgggaacgg
aaaaattattttattaaagagtagttcaacaaacgggccagtttgttgaagattagatgctataattgttatt
aaaaggattgaaggatgggag

pCasPP_spo0A-SNP

ctgttacaggcatattcatatcaatgtcgccatcgatggaaataatttcaatggcatattctctaaaaaattt
gtatttagggctagaatttagaaagaaaatgtattattatagaagaggtagaacattaaggaatgatgtgaaa
gtgctagcactgaatgatagccttgctaataagtctatcatctggacacacaggatgatcttggttcatgtta
gcaccttttaggcaatgaatctactaaacggcagccgtttaggatcattgtcttaaagtttcataaaaacgaa
gggggattttaggcttttacttaatggacaagaagtacagcatcggcctggacatcggcaccaacagcgtggg
ctgggcggtcatcaccgacgagtacaaggtcccctccaagaagttcaaggtcctgggcaacaccgaccggcac
tcgatcaagaagaacctgatcggcgccctgctcttcgacagcggcgaaaccgccgaggcgacccgcctgaagc
ggaccgcccgtcgccgctacacccggcgcaagaaccgcatctgctacctgcaggagatcttctccaacgagat
ggccaaggtcgacgactcgttcttccaccggctcgaggagagcttcctggtggaggaggacaagaagcacgag
cgccacccgatcttcggcaacatcgtcgacgaggtggcctaccacgagaagtaccccaccatctaccacctcc
gcaagaagctggtcgactcgaccgacaaggcggacctgcggctcatctacctggccctcgcgcacatgatcaa
gttccgcggccacttcctcatcgagggcgacctgaacccggacaactccgacgtcgacaagctcttcatccag
ctggtgcagacctacaaccagctgttcgaggagaaccccatcaacgccagcggcgtcgacgccaaggcgatcc
tctccgcgcgcctgagcaagtcccggcgcctggagaacctcatcgcccagctgccgggcgagaagaagaacgg
cctcttcggcaacctgatcgcgctgtcgctcggcctgacccccaacttcaagagcaacttcgacctggccgag
gacgcgaagctccagctgtccaaggacacctacgacgacgacctggacaacctgctcgcccagatcggcgacc
agtacgcggacctcttcctggccgcgaagaacctctcggacgccatcctgctcagcgacatcctgcgggtcaa
caccgagatcaccaaggccccgctgtcggcgagcatgatcaagcggtacgacgagcaccaccaggacctgacc
ctgctcaaggccctcgtgcgccagcagctgcccgagaagtacaaggagatcttcttcgaccagtccaagaacg
gctacgccggctacatcgacggcggcgcgtcgcaggaggagttctacaagttcatcaagccgatcctggagaa
gatggacggcaccgaggagctgctcgtcaagctgaaccgcgaggacctgctccgcaagcagcggaccttcgac
aacggctccatcccgcaccagatccacctgggcgagctccacgccatcctccggcgccaggaggacttctacc
ccttcctgaaggacaaccgcgagaagatcgagaagatcctgaccttccggatcccgtactacgtcggcccccct
ggcccgcggcaactcccggttcgcgtggatgacccggaagtcggaggaaaccatcaccccgtggaacttcgag
gaggtcgtggacaagggcgcctccgcgcagtcgttcatcgagcgcatgaccaacttcgacaagaacctcccga
acgagaaggtcctgcccaagcacagcctgctctacgagtacttcaccgtgtacaacgagctgaccaaggtcaa
gtacgtgaccgagggcatgcggaagccggccttcctgtccggcgagcagaagaaggcgatcgtcgacctgctc
ttcaagaccaaccgcaaggtcaccgtgaagcagctgaaggaggactacttcaagaagatcgagtgcttcgact
ccgtcgagatctcgggcgtggaggaccgcttcaacgcctccctgggcacctaccacgacctgctcaagatcat
caaggacaaggacttcctcgacaacgaggagaacgaggacatcctggaggacatcgtcctcaccctgaccctc
ttcgaggaccgcgagatgatcgaggagcggctcaagacctacgcccacctgttcgacgacaaggtgatgaagc
agctgaagcggcgccggtacaccggctggggccgcctctcccggaagctgatcaacggcatccgggacaagca
gagcggcaagaccatcctggacttcctcaagtccgacggcttcgccaaccgcaacttcatgcagctcatccac
gacgactcgctgaccttcaaggaggacatccagaaggcccaggtgtccggccagggcgacagcctccacgagc
acatcgccaacctggcgggctccccggcgatcaagaagggcatcctccagaccgtcaaggtcgtggacgagct
ggtcaaggtgatgggccgccacaagcccgagaacatcgtgatcgagatggcccgggagaaccagaccacccag
aagggccagaagaactcccgcgagcggatgaagcgcatcgaggagggcatcaaggagctcggctcgcagatcc
tgaaggagcacccggtcgagaacacccagctccagaacgagaagctgtacctctactacctgcagaacggccg
cgacatgtacgtggaccaggagctcgacatcaaccggctgagcgactacgacgtcgaccacatcgtgccgcag
tccttcctgaaggacgactcgatcgacaacaaggtcctgacccgctccgacaagaaccgggggcaagtccgaca
acgtgccctcggaggaggtcgtgaagaagatgaagaactactggcgccagctgctcaacgccaagctcatcac
ccagcgcaagttcgacaacctgaccaaggccgagcggggcggcctgtcggagctcgacaaggcgggcttcatc
aagcgccagctcgtcgaaacccggcagatcaccaagcacgtggcccagatcctggacagccggatgaacacca

agtacgacgagaacgacaagctgatccgcgaggtcaaggtgatcaccctcaagagcaagctggtgtccgactt
ccgcaaggacttccagttctacaaggtccgggagatcaacaactaccaccacgcccacgacgcgtacctgaac
gccgtcgtgggcaccgcgctgatcaagaagtacccgaagctggagtccgagttcgtctacggcgactacaagg
tctacgacgtgcgcaagatgatcgccaagtcggagcaggagatcggcaaggccaccgcgaagtacttcttcta
cagcaacatcatgaacttcttcaagaccgagatcaccctggccaacggcgagatccgcaagcggcccctgatc
gaaaccaacggcgaaaccggcgagatcgtctgggacaagggccgcgacttcgccaccgtccggaaggtgctgt
ccatgccgcaggtcaacatcgtcaagaaaaccgaggtgcagaccggcggcttcagcaaggagtccatcctccc
caagcgcaactcggacaagctgatcgcccggaagaaggactgggacccgaagaagtacggcggcttcgacagc
cccaccgtcgcctactccgtgctggtcgtggcgaaggtcgagaagggcaagagcaagaagctgaagtccgtga
aggagctgctcggcatcaccatcatggagcgctcctcgttcgagaagaacccgatcgacttcctggaggccaa
gggctacaaggaggtcaagaaggacctcatcatcaagctgcccaagtactcgctgttcgagctcgagaacggc
cgcaagcggatgctcgccagcgcgggcgagctgcagaagggcaacgagctggccctcccgtccaagtacgtca
acttcctgtacctcgcgtcccactacgagaagctgaagggctcgcccgaggacaacgagcagaagcagctctt
cgtggagcagcacaagcactacctggacgagatcatcgagcagatctcggagttcagcaagcgggtcatcctg
gccgacgcgaacctcgacaaggtgctgtccgcctacaacaagcaccgcgacaagccgatccgggagcaggcgg
agaacatcatccacctgttcaccctcaccaacctgggcgcccccgccgcgttcaagtacttcgacaccaccat
cgaccgcaagcggtacaccagcaccaaggaggtcctcgacgcgaccctgatccaccagtccatcaccggcctg
tacgaaacccgcatcgacctctcccagctcggcggcgactgagaattcagatctacgcgttccccgcaaaagc
ggcctttgactccctgcaagcctcagcgaccgaatatatcggttatgcgtgggcgatggttgttgtcattgtc
ggcgcaactatcggtatcaagctgtttaagaaattcacctcgaaagcaagctgataaaccgatacaattaaag
gctccttttggagcctttttttgctgctccttcggtcggacgtgcgtctacgggcaccttaccgcagccgtcg
gctgtgcgacacggacggatcgggcgaactggccgatgctgggagaagcgcgctgctgtacggcgcgcaccgg
gtgcggagcccctcggcgagcggtgtgaaacttctgtgaatggcctgttcggttgctttttttatacggctgc
cagataaggcttgcagcatctgggcggctaccgctatgatcggggcgttcctgcaattcttagtgcgagtatc
tgaaaggggatacgctaagctgagaattgagaacagttttagagctagaaatagcaagttaaaataaggctag
tccgttatcaacttgaaaaagtggcaccgagtcggtgctttttactccatctggatttgttcagaacgctcgg
ttgccgccgggcgtttttttatctagctagacctgacttccgcctgcagggccagctcgcggacgtgctcatag
tccacgacgcccgtgattttgtagccctggccgacggccagcaggtaggccgacaggctcatgccggccgccg
ccgcctttttcctcaatcgctcttcgttcgtctggaaggcagtacaccttgataggtgggctgcccttcctggt
tggcttggtttcatcagccatccgcttgccctcatctgttacgccggcggtagccggccagcctcgcagagca
ggattcccgttgagcaccgccaggtgcgaataagggacagtgaagaaggaacacccgctcgcgggtgggccta
cttcacctatcctgcccggctgacgccgttggatacaccaaggaaagtctacacgaaccctttggcaaaatcc
tgtatatcgtgcgaaaaaggatggatataccgaaaaaatcgctataatgaccccgaagcagggttatgcagcg
gaaaagatccgtcgacctgcatactagctgctcaaggtcgcccgcaaccggcgcatcaagcccgccgcgacag
aaggaattcataattcgatgtcgaaatcatatactccgacaagaaatatttatatactgtaaacagatatcac
aatggaatatcactcaatgaggaggaagtacattgcaaaaaattgaggtattgttggctgatgacaaccggga
atttacgaatttgcttgctgaatatatttccgatcaggaggatatggaagttacaggaatcgcctataacggc
gaagaagtgctccaacgcatcgcagaatcccgcaacatacctgatgtacttattttagatattattatgcctc
atctggatggtctcggtgtattggagcgtttaagagaaatgaacctgactccacagccgaaaatcattatgct
gactgcattcggtcaagaaaatattacacagagggccgtacagctcggggcatcttattatatttgaagccg
tttgacatggaagtgctggctaaccgtgttcgccaattagtggggccgcagttagttagcagcagtccggtga
ccatttcatccatgcggtccaacgttgtacctatgggtaagacgaaaaacctggatgccagtatcacggccat
catacatgaaattggtgtaccagctcacatcaagggctatcaatatttacgcgaagccattacgatggtgtac
aataatatcgaaatttgggagccatcaccaaaacattatatccggcaatcgccgaaaagttcaaaacgacgg
catctcgcgtagaacgcgccattcgtcatgctatcgaggtagcatggacccgtggaaatatcgacagcatttc
acacctgttcgggtacaccattaatatcagtaagtccaaaccaaccaattcagagttcatagcgatggtagta
gataaactaaggattgagaacaaagtgtcctgaaagggtcagaatgattgatgaagcaagggattttgtggt
tttacgtttatcaatgataattgcagggatggctattaatttccaaaacagccgacccgatccgataaacttc
tgaatagtagacaactaataccgcctatttattggttcttccaagaaccagtaaataggcggtatttttata
aacgattttgaatttcaattggacaatttaaagtttcttcaattatcatcattttcattactttcagtagtc
gttctattaaggtagaaagttgtaaagaaatattattgaactattgcttgatcgacaagctctattagaaata
taattaaaacaatctttcaaatgaggtgaatcggatgtctttccgtattgttcgaggtatttagtttccgggc
tggaaaaaacgcgtaccagccgtcaagggaaagtctattcttttgactacaaaactaaaatactgaatgata
ttggtctgctatcccgtagtatttatccttaattggaaggtggaaagaaaatgaattcaatagagtataaaga
gttttataataagattggaaaaatcaacggctggaatttcagcaatgtgaaatgtatctccaaaggagtaaaa
tgggacttctacaatgaagtagcgaagacatgcaagaagtcggatatattgcttgacatcggtactggcggcg
gggaagcaattttatcaatagcagattctgcattactttagttgggattgaccattccaccggaatgattga
aacagcaaccaagaattctgctgaatcagacatagcaaatgttcgttttctccagatggatgctgaaaatcta
aattttcctgagaacttctttaatgtaatctcttctcgacattcatgtttttatgcaaaagaaatagcaaagg
tgttagtaaaggatggtatgttcctgacacagcaagtaagcgaaaacgacaaattaaatattaaagaagcgtt
tggaagaggacaggcatgggccaggaaccgtaaaaaggccgcgttgctggcgtttttccataggctccgcccc

```
cctgacgagcatcacaaaaatcgacgctcaagtcagaggtggcgaaacccgacaggactataaagataccagg
cgtttccccctggaagctccctcgtgcgctctcctgttccgaccctgccgcttaccggatacctgtccgcctt
tctcccttcgggaagcgtggcgctttctcatagctcacgctgtaggtatctcagttcggtgtaggtcgttcgc
tccaagctgggctgtgtgcacgaaccccccgttcagcccgaccgctgcgccttatccggtaactatcgtcttg
agtccaacccggtaagacacgacttatcgccactggcagcagccactggtaacaggattagcagagcgaggta
tgtaggcggtgctacagagttcttgaagtggtggcctaactacggctacactagaagaacagtatttggtatc
tgcgctctgctgaagccagttaccttcggaaaaagagttggtagctcttgatccggcaaacaaaccaccgctg
gtagcggtggtttttttgtttgcaagcagcagattacgcgcagaaaaaaaggatctcaagaagatcctttgat
cttttctacggggtctgacgctcagtggaacgaaaactcacgttaagggattttggtcatgagattatcaaaa
aggatcttcacctagatccttttggttcatgtgcagctccatcagcaaaaggggatgataagtttatcaccac
cgactatttgcaacagtgccgttaatggttataatagctgaataagaacggtgctctccaaatattcttattt
agaaaagcaaatctaaaattatctgaaaaggaatgagaatagtgaatggaccaataataatgactagagaag
aaagaatgaagattgttcatgaaattaaggaacgaatattggataaatatggggatgatgttaaggctattgg
tgtttatggctctcttggtcgtcagactgatgggccctattcggatattgagatgatgtgtgtcatgtcaaca
gaggaagcagagttcagccatgaatggacaaccggtgagtggaaggtggaagtgaattttgatagcgaagaga
ttctactagattatgcatctcaggtggaatcagattggccgcttacacatggtcaatttttctctattttgcc
gatttatgattcaggtggatacttagagaaagtgtatcaaactgctaaatcggtagaagcccaaacgttccac
gatgcgatttgtgcccttatcgtagaagagctgtttgaatatgcaggcaaatggcgtaatattcgtgtgcaag
gaccgacaacatttctaccatccttgactgtacaggtagcaatggcaggtgccatgttgattggtctgcatca
tcgcatctgttatacgacgagcgcttcggtcttaactgaagcagttaagcaatcagatcttccttcaggttat
gaccatctgtgccagttcgtaatgtctggtcaactttccgactctgagaaacttctggaatcgctagagaatt
tctggaatgggattcaggagtggacagaacgacacggatatatagtggatgtgtcaaaacgcataccattttg
aacgatgacctctaataattgttaatcatgttggttacgtatttattaacttctcctagtattagtaattatc
atggctgtcatggcgcattaacggaataaagggtgtgcttaaatcgggccattttgcgtaataagaaaaagga
ttaattatgagcgaattgaattaataataaggtaatagatttacattagaaaatgaaaggggattttatgcgt
gagaatgttacagtctatcccggcattgccagtcggggatattaaaaagagtataggtttttattgcgataaa
ctaggtttcactttggttcaccatgaagatggattcgcagttctaatgtgtaatgaggttcggattcatctat
gggaggcaagtgatgaagctggcgctctcgtagtaatgattcaccggtttgtacaggtgcggagtcgtttatt
gctggtactgctagttgccgcattgaagtagagggaattgatgaattatatcaacatattaagcctttgggca
ttttgcaccccaatacatcattaaaagatcagtggtgggatgaacgagactttgcagtaattgatcccgacaa
caatttgattagctttttttcaacaaataaaaagctaaaatctattattaatctgttcagcaatcgggcgcgat
tgctgaataaaagatacgaaggtgatggttttgaacttgttctttcttatcttgatacatatagaaataacgt
catttttattttagttgctgaaaggtgcgttgaagtgttggtatgtatgtgttttaaagtattgaaaaccctt
aaaattggttgcacagaaaaaccccatctgttaaagttataagtgactaaacaaataactaaatagatggggg
tttcttttaatattatgtgtcctaatagtagcatttattcagatgaaaaatcaagggttttagtggacaagac
aaaaagtggaaaagtgaggccatggagagaaaagaaaatcgctaatgttgattactttgaacttctgcatatt
cttgaatttaaaaaggctgaaagagtaaaagattgtgctgaaatattagagtataaacaaaatcgtgaaacag
gcgaaagaaagttgtatcgagtgtggttttgtaaatccaggctttgtccaatgtgcaactggaggagagcaat
gaaacatggcattcagtcacaaaaggttgttgctgaagttattaaacaaaagccaacagttcgttggttgttt
ctcacattaacagttaaaaatgtttatgatggcgaagaattaaataagagtttgtcagatatggctcaaggat
ttcgccgaatgatgcaatataaaaaaattaataaaaatcttgttggttttatgcgtgcaacggaagtgacaat
aaataataaagataattcttataatcagcacatgcatgtattggtatgtgtggaaccaacttattttaagaat
acagaaaactacgtgaatcaaaaacaatggattcaattttggaaaaaggcaatgaaattagactatgatccaa
atgtaaaagttcaaatgattcgaccgaaaaataaatataaatcggatatacaatcggcaattgacgaaactgc
aaaatatcctgtaaaggatacggattttatgaccgatgatgaagaaaagaatttgaaacgtttgtctgatttg
gaggaaggtttacaccgtaaaaggttaatctcctatggtggtttgttaaaagaaatacataaaaaattaaacc
ttgatgacacagaagaaggcgatttgattcatacagatgatgacgaaaaagccgatgaagatggattttctat
tattgcaatgtggaattgggaacggaaaaattattttattaaagagtagttcaacaaacgggccagtttgttg
aagattagatgctataattgttattaaaaggattgaaggatggag
```
